# Molecular Characterization and Expression Profiling of NAC Transcription Factors in *Brachypodium distachyon* L

**DOI:** 10.1371/journal.pone.0139794

**Published:** 2015-10-07

**Authors:** Gengrui Zhu, Guanxing Chen, Jiantang Zhu, Yan Zhu, Xiaobing Lu, Xiaohui Li, Yingkao Hu, Yueming Yan

**Affiliations:** College of Life Science, Capital Normal University, Beijing, 100048, China; Julius Kuehn-Institute (JKI), GERMANY

## Abstract

NAC (NAM, ATAF1/2, CUC2) transcription factors are involved in regulating plant developmental processes and response to environmental stresses. *Brachypodium distachyon* is an emerging model system for cereals, temperate grasses and biofuel crops. In this study, a comprehensive investigation of the molecular characterizations, phylogenetics and expression profiles under various abiotic stresses of the NAC gene family in *Brachypodium distachyon* was performed. In total, 118 *BNAC* genes in *B*. *distachyon* were identified, of which 22 (18.64%) were tandemly duplicated and segmentally duplicated, respectively. The Bayesian phylogenetic inference using Markov Chain Monte Carlo (MCMC) algorithms showed that they were divided into two clades and fourteen subfamilies, supported by similar motif compositions within one subfamily. Some critical amino acids detected using DIVERGE v3.0 might contribute to functional divergence among subfamilies. The different exon-intron organizations among subfamilies revealed structural differentiation. Promoter sequence predictions showed that the *BNAC* genes were involved in various developmental processes and diverse stress responses. Three NAC domain-encoding genes (*BNAC012*, *BNAC078 and BNAC108*), orthologous of *NAC1*, were targeted by five miRNA164 (Bdi-miR164a-c, e, f), suggesting that they might function in lateral organ enlargement, floral development and the responses to abiotic stress. Eleven (~9.32%) BNAC proteins containing α-helical transmembrane motifs were identified. 23 representative *BNAC* genes were analyzed by quantitative real-time PCR, showing different expression patterns under various abiotic stresses, of which 18, 17 and 11 genes were up-regulated significantly under drought, H_2_O_2_ and salt stresses, respectively. Only four and two genes were up-regulated under cold and cadmium stresses, respectively. Dynamic transcriptional expression analysis revealed that six genes showed constitutive expression and period-specific expression. The current results provide novel insights into the structure and function of the plant NAC gene family.

## Introduction

Adverse stresses affect biomass and agricultural productivity worldwide significantly due to the deterioration of the global environment [1–3]. However, plants have developed numerous physiological and biochemical strategies to protect cellular activities and maintain plant integrity to ensure their survival under adverse conditions [3,4]. Transcription factors (TFs) regulate the expression of stress-related genes by binding to the cognate *cis*-acting elements [5,6] that control all biological processes in plants, including growth, development and regulating the gene responses to developmental and environmental changes [7]. However, the plant-specific NACTF family, is known for their broad roles in several developmental programs, defence and stress-responses [8,9].

Since the first comprehensive review of NAC TFs in 2005 [8], much research has provided us more knowledge about NAC transcription factors. The name NAC was derived from the three mutants earliest identified: NAM from petunia [10], and ATAF1-2 and CUC from *Arabidopsis* [11]. Typically, NAC proteins have two parts: a conserved N-terminal NAC domain and C-terminal variable transcription regulatory regions (TRRs) [8]. The N-terminal NAC domain (~160 amino acids) consists of subdomains A–D (NAM domain in InterPro) and an additional subdomain E [12].Generally, subdomains A, C and D are highly conserved, of which subdomain A may be involved in the formation of a functional dimer and subdomains C and D bind to DNA, whereas subdomains B and E are divergent [9,12].It was reported that subdomain A–E in typical NAC proteins contain five motifs, whereas other distinct motifs form NAC-like proteins [13]. In addition to the N-terminal NAC domain, the C-terminal TRRs operate as functional domains by conferring either activation or repression activity [14]. However, some NAC TFs contain α-helical transmembrane (TM) motifs at their C terminus, which are responsible for anchoring to the plasma membrane or endoplasmic reticulum [15]. The *Arabidopsis* and rice genomes contain at least 85 and 45 membrane-bound transcription factors (MTFs), respectively. Among these, at least 18 NAC MTFs are present in *Arabidopsis* and five are expressed in rice [16]. These NAC MTFs are classified as membrane-related and named NTL (NTM1-like or ‘NAC with transmembrane motif 1’-Like) TFs [14].

The activity of *NAC* genes is regulated through three processes: the binding of specific TFs to regulatory promoter regions at the transcriptional level, miRNA164-mediated cleavage or alternatively splicing at the post-transcriptional level and ubiquitins, dimerization and/or interaction with other non-NAC proteins at the post-translational level [9]. These regulatory steps control the involvement of NAC TFs in plant developmental processes [10,17–19] as well as the responses to biotic and abiotic stresses [1,6,20]. To date, the function of numerous *NAC* genes has been verified using transgene technologies or microarrays [9,21]. The overexpression of three *Arabidopsis thaliana* NAC genes (*ANAC019*, *ANAC055* and *ANAC072/RD26*) in transgenic plants increased the stress tolerance of the plants compared with the wild type plants [6]. *Arabidopsis* plants overexpressing *ATAF1* are highly sensitive to abscisic acid (ABA), high-salt, oxidative stress and necrotrophic fungus (*B*. *cinerea*) [22]. *OsNAC6*, the rice homolog of *ATAF1*, has a higher tolerance to drought and high salinity [23].In soybean, GmNAC5, a member of CUC/NAM subfamily, plays a role in seed germination and abiotic stress responses [24]. In transgenic *Arabidopsis* lines, the overexpression of *TaNAC2* enhances the tolerance to drought, cold and salt stresses [3]. Nevertheless, revealing their roles in abiotic stresses remains challenging in view of their large numbers and various functions under complex environmental stimuli [21].

To date, the *NAC* gene family has been investigated and identified in *Arabidopsis*, rice and some other higher plants [12–14,25–27]. However, comprehensive investigations of the NAC family in *Brachypodium distachyon* have been less reported. A scanty report was published about BdSWN5 TF, which regulates a secondary cell-wall cellulose synthase (*BdCESA4*), a xylem-specific protease (*BdXCP1*) and an orthologous of AtMYB46 (*BdMYB1*), suggesting that it is capable of turning on secondary cell-wall synthesis and cell death programs [28]. *B*. *distachyon* has rapidly become a powerful model system for cereals, temperate grasses and biofuel crops [29]. Furthermore, the recent availability of a high-quality sequencing of the *B*. *distachyon* genome [30] provides an unprecedented opportunity for genome-wide analysis of all genes of the NAC gene family. Therefore, research into the NAC TFs in *B*. *distachyon* species will contribute to future studies of agriculturally important monocots.

In this study, we performed a comprehensive investigation of the molecular characterizations, phylogenetics and expression profiles of NAC genes in *B*. *distachyon* under various abiotic stresses. The results provide novel insights into the plant NAC genes and their molecular mechanisms of action in response to adverse environments.

## Materials and Methods

### Sequence retrieval and identification

To roundly collect members of NAC gene family in *B*. *distachyon*, the protein sequences of the published *Arabidopsis thaliana* NAC (ANAC) and *Oryza sativa* NAC (ONAC) [12,25] were used to BLASTP program against phytozome v10.2 (http://phytozome.jgi.doe.gov/pz/portal.html). The protein up to E value ≤ 1E-10 was selected as candidate protein, and was excluded if its amino acid sequence < 100 residues. Each annotated protein was confirmed a Hidden Markov Model (HMM) profile of the NAM domain PF02365 by Pfam (http://pfam.xfam.org/) searches and checked for the existence of NAC domain by SMART (http://smart.embl-heidelberg.de/).

### Chromosomal locations, gene duplication analyses of *NAC* genes in *B*. *distachyon*

Locations of *BNAC* genes on the *Brachypodium* chromosome maps obtained from Phytozome v10.2 were mapped by MapInspect program and modified manually. Furthermore, tandem duplicated genes were defined as adjacent homologous genes on a single chromosome, with no more than one intervening gene. Segmental duplication was collected using the Plant Genome Duplication Database (PGDD, http://chibba.agtec.uga.edu/duplication/) with the range of 100kb and the gene pairs, of which synonymous substitution rates (Ks) values were between 0 and 1.0, were selected to calculate the dates of duplication events (T) using the mean Ks values. The dates were calculated by the equation T = Ks/2λ, assuming clock-like rates of synonymous substitution of 6.5 × 10^−9^ substitutions/synonymous site/year for *Brachypodium* [31].

### Estimates of functional divergence

The functional divergence between pairwise subfamilies of the NAC proteins was analyzed using the software DIVERGE v3 [32]. θ-I and θ-II, the coefficients of Type-I and Type-II functional divergence, were calculated between pairwise clusters of the family. If θ-I or θ-II is significantly greater than 0, it means that site specific altered selective constraints or a radical shift of amino acid physiochemical property occurred after gene duplication and/or speciation. Furthermore, critical amino acid residues were predicted based on posterior probability (Qk). Qk > 0.9, as a threshold, was to screen potentially crucial sites for functional divergence [32].

### Phylogenetics and molecular characterization

Phylogenetic trees were constructed based on Bayesian inference using Markov Chain Monte Carlo (MCMC) methods [33]. Initially, the NAC amino acid sequences of the whole coding regions were aligned by using the MUSCLE program with default parameters. Then bayesian inference phylogenetic construction was performed with MrBayes v 3.2 using GTR (General Time Reversible) model with gamma distributed rates (gamma-distributed rate variation) [34]. Bayesian analysis included mcmcngen = 10^6^ and samplefreq = 10. When the average standard deviation of split frequencies was below 0.01, the operation was terminated. After discarding the burn-in samples (first 25% of samples), the remaining data were used to generate a Bayesian tree, which was presented by software FigTree v1.4.2. The motifs of NAC proteins were identified using MEME v4.10.1 (Multiple Em for Motif Elicitation, http://meme-suite.org/index.html). The exon/intron organizations were derived from Gene Structure Display Server (GSDS, http://gsds.cbi.pku.edu.cn/). The protein pI/Mw was determined by the Compute pI/Mw tool (http://web.expasy.org/compute_pi/). The 1500 bp upstream sequences as promoter regions were collected from Phytozome and submitted to PlantCARE database (http://bioinformatics.psb.ugent.be/webtools/plantcare/html/) to search their putative *cis*-acting elements. The miRBase (http://www.mirbase.org/search.shtml) was used to search identified miRNA164s, then the secondary structures and potential targets were predicted using the RNAfold web server (http://rna.tbi.univie.ac.at/cgi-bin/RNAfold.cgi) and psRNATarget (http://plantgrn.noble.org/psRNATarget/), respectively. The predictions of membrane-bound BNAC proteins were determined by TMHMM server v.2.0 (http://www.cbs.dtu.dk/services/TMHMM/).

### Plant materials, growth conditions and stress treatments

The uniform seeds of standard diploid inbred line of Bd21 were sterilized with 75% alcohol and 15% sodium hypochlorite and then washed three times with sterile water. The seeds were germinated on filter paper saturated with water in complete darkness at 26°C. After 3 days, seedlings were grown in Hoagland solution in the greenhouse under a 16/8-h (light/dark) photocycle at 28/26°C (day/night). The nutrient solution was changed every 3 days. The 2-week-old seedlings were incubated in the following conditions: 200 mM NaCl (salinity stress), 4°C (cold stress), PEG6000 (mild drought stress), 500 uM CdCl_2_ (heavy metal stress) and 20 mM H_2_O_2_ (oxidative stress). Leaves of control and treated seedlings were harvested at 6, 12, 24 and 48h for assays. All samples were immediately stored at -80°C immediately until used.

### RNA extraction and qRT-PCR

Total RNA was extracted using TRIzol reagent (Invitrogen) based on the published manufacturer’s instructions. PrimeScript™ RT Master Mix (Perfect Real Time) was used for RNA purification and reverse transcription following the manufacturer’s instructions. For RT-PCR, specific primer pairs (S1 Table) were designed using the Primer3Plus program (http://www.bioinformatics.nl/cgi-bin/primer3plus/primer3plus.cgi). The designed primer pairs were checked by Primer-BLAST tool in NCBI database (http://www.ncbi.nlm.nih.gov/tools/primer-blast/index.cgi?LINK_LOC=BlastHome) to confirm the consistency and uniqueness of them. Ubi4 (Bradi3g04730) was selected as the reference gene according to previous report [35]. The qRT-PCR programs were performed in three biological replicates by a CFX96 Real-Time PCR Detection System (Bio-Rad) using 2^-△△^Ct method with the following parameters: an initial denaturation step at 95°C for 3 min, followed by three procedures: denaturation at 95°C for 15s, anneal at 60°C for 15s, extension at 72°C for 20s, in total of 40 cycles. Fluorescence was measured at the end of each cycle. The qRT-PCR efficiency was determined by five serial five-fold dilutions of cDNA, the standard curve showed the RT-PCR efficiency rate and melting curves of *BNAC* genes showed single peaks (S1 File).

## Results

### *In silico* identification and annotation of the NAC gene family in *B*. *distachyon*

A total of 115 NAC sequences from *Arabidopsis* and 141 from *Oryza sativa* were used separately to perform a BLAST search for *Brachypodium* NAC family genes, and a list of 118 *NAC* genes of *Brachypodium distachyon* were identified and used for further analyses (S2 Table). Based on the *Arabidopsis* and rice NAC nomenclature suggestions and to distinguish from existing alias, each gene was named as following: an initial letter corresponding to *B*. *distachyon* (B), followed by the family designation (NAC) and a number, as shown in S3 Table. Among higher plants, the dicot species *Arabidopsis* is a model plant used to predict the function of unknown genes, and comparative genomic analysis of the NAC family between *Brachypodium* and *Arabidopsis* allowed the functions of several *BNAC* genes to be deduced from their *Arabidopsis* orthologous. Several BNAC genes share an *Arabidopsis* orthologous, for example, BNAC030 and BNAC040 are orthologous of ATAF1 with strong e-value support. Interestingly, *BNAC* genes belonging to subgroups VII–IX, and XI–XIV showed low E-value with corresponding *Arabidopsis* orthologous, whereas genes in subgroup X exhibited higher orthologous with *ANAC* genes (S3 Table).

The genetic characteristics and loci of the *BNAC* genes are summarized in S3 Table. The lengths of the proteins ranged from 106 to 856 amino acids, with molecular weights of 11665.5 to 96519.0 kDa. The pI values of 4.16–10.06, did not correspond to their masses. These results indicated that *BNAC* genes were not conserved during evolution.

### Chromosomal distribution and gene duplication events among *BNAC* genes

**Fig 1:**
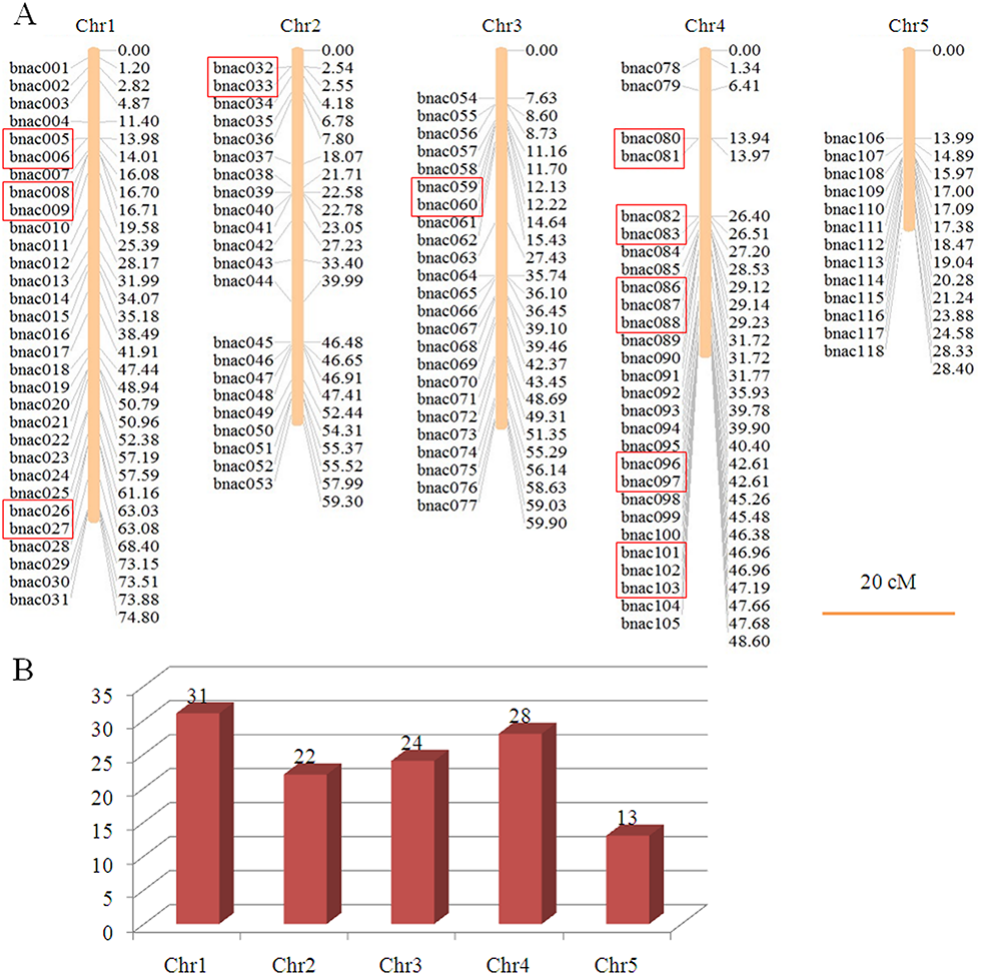
Distribution of *BNAC* genes on five *Brachypodium* chromosomes. (A) The size of each chromosome reflects its relative length and the chromosome numbers are indicated at the top of each bar. The red boxes represent tandem duplicated genes. The figure was produced using the MapInspect software. The scales are shown in megabases (Mb). (B) The percent of *BNAC* genes on each chromosome shows their distribution abundance.

The locations of *BNAC* genes on the five chromosomes of *B*. *distachyon* were shown in Fig 1A, members of the BNAC family were distributed non-randomly on the five chromosomes. As shown in Fig 1B, chromosome 1 contained the largest number (31, ~26.3%) of *BNAC* genes, followed by chromosome 4, which contained 28 members (~23.7%). Chromosome 5 contained only 13 members (~11.0%). The precise chromosome position of each *BNAC* gene is shown in S3 Table. However, the gene numbers on each chromosome were directly proportional to the length of the corresponding chromosome, except for chromosome 4 (Fig 1A and 1B), suggesting that *NAC* genes in *Brachypodium* have no obvious chromosomal preferences. Furthermore, the genes were prone to being distributed in clusters at certain chromosomal regions, especially in the chromosome 4 and 5, and were dispersed in a single manner at other locations (Fig 1A), consistent with other plants [27,28].

Segmental duplication, tandem duplication and transposition events (retroposition and replicative transposition) are the three dominant evolutionary patterns [36], of which segmental duplication and tandem duplication, resulting from polyploidy and unequal crossing-over, respectively, are common in plants [37]. The current study identified the ones that tandem duplicated genes and segment duplicated genes both accounted for 18.64% (22 of 118). As shown in Fig 1A, the tandem duplicated genes were distributed on chromosomes 1, 2, 3 and 4, whereas chromosomes 1–5 contained segmentlly duplicated genes. Obviously, two groups (*BNAC086*, *BNAC087*, *BNAC088* and *BNAC101*, *BNAC102*, *BNAC103*) happened that three genes were involved in tandem duplication events. The mean Ks values were determined to estimate the dates of the segmental duplication events (Table 1). These events happened mainly during 45.38–73.05 million years ago (MYA). Almost half segmental duplications (five of eleven pairs) occurred on the same chromosome. This suggests that both segmental duplication and tandem duplication is the major expansion pattern of the BNAC gene family in *Brachypodium*.

**Table 1:** Estimates of the dates for the segmental events between the duplicated *BNAC* genes.

Segment pairs	Numbers of anchors	Ks (mean ± s.d.)	Estimated time (mya)
BNAC005&BNAC027	4	0.69 ± 0.034	53.08
BNAC006&BNAC026	4	0.69 ± 0.034	53.08
BNAC015&BNAC054	4	0.83 ± 0.079	63.85
BNAC049&BNAC105	3	0.79 ± 0.131	60.77
BNAC041&BNAC052	3	0.72 ± 0.253	55.38
BNAC040&BNAC051	5	0.59 ± 0.176	45.38
BNAC071&BNAC057	5	0.60 ± 0.159	46.15
BNAC072&BNAC110	5	0.79 ± 0.164	60.77
BNAC038&BNAC050	7	0.65 ± 0.190	50
BNAC058&BNAC118	4	0.95 ± 0.058	73.08
BNAC073&BNAC114	8	0.75 ± 0.188	57.69

MYA: million years ago

λ = 6.5×10^−9^

### Phylogenetic and structural analysis of the BNAC family

**Fig 2:**
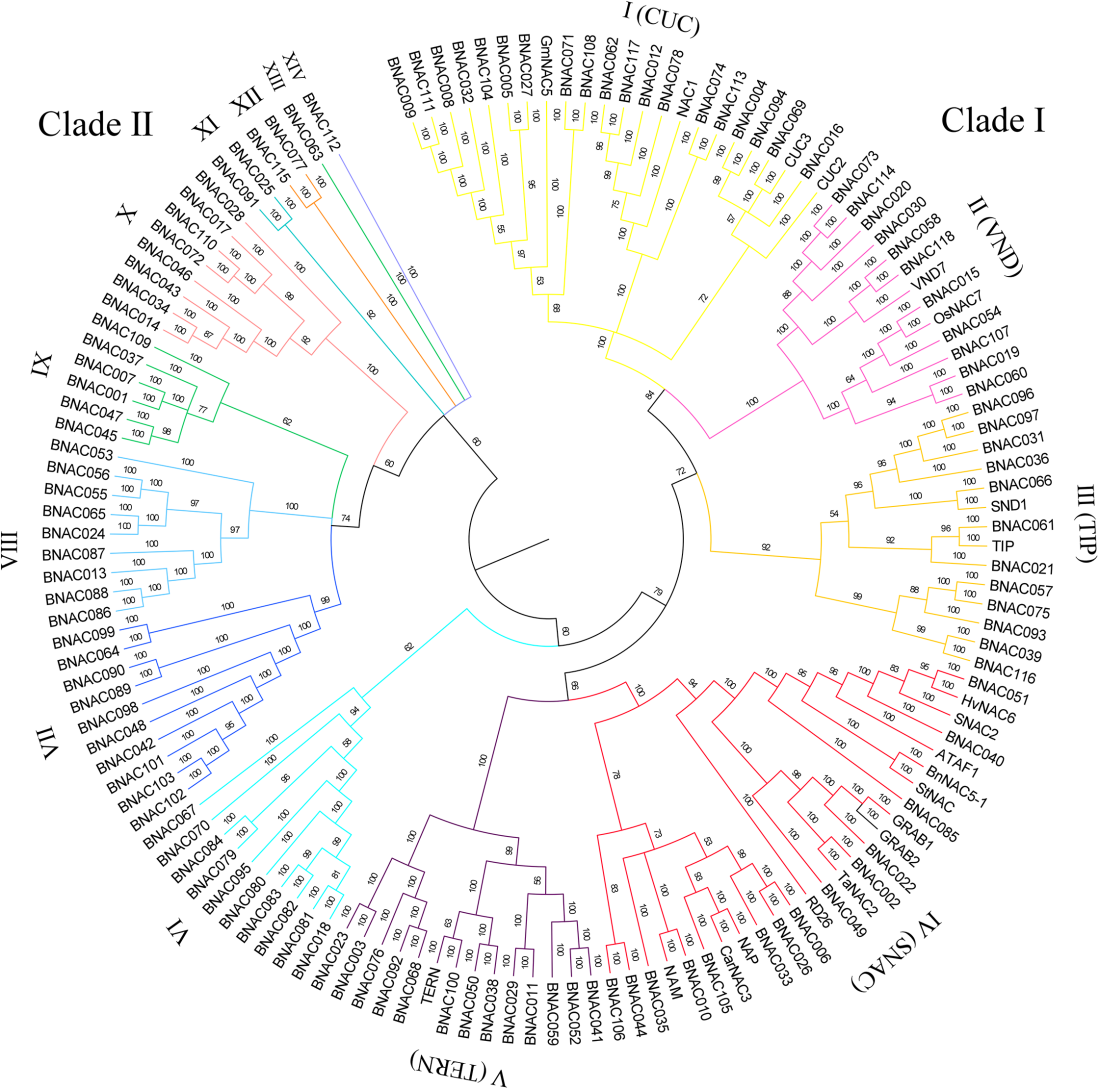
Phylogenetic relationships of BNAC proteins. The amino acid sequences were aligned using MUSCLE program and the Bayesian tree was generated by MrBayes v 3.2, using MCMC algorithms and GTR model with gamma distributed rates. Each subfamily is highlighted in a different color.

The Bayesian inference, implemented by MrBayes, has become a standard approach for the estimation of branch support as posterior probabilities within the time required for the run [33,38]. To reveal the phylogenetic relationships of the BNAC family and predict the functions of certain subfamilies, we have implemented a Bayesian MCMC algorithm to infer the phylogenetic tree. Finally the tree was classified into fourteen subgroups, of which five subgroups were named with their orthologous groups namely, CUC (development-related NAC), VND (secondary wall-synthesis NAC), TIP (membrane-associated NAC), TERN (tobacco elicitor-responsive NAC) and SNAC (stress-related NAC), since they may be involved in similar regulatory roles. As displayed schematically in S1 and S2 Figs, ten types of motifs were detected, including seven types of NAC motifs, corresponding to five NAC subdomains. Despite the great variations in the motifs among the various subgroups VI–XIV (S1 Fig), the members clustered in the same subgroup shared similar motif compositions, which supported our classifications (Fig 2). The members of the subgroups I–V had comparatively higher motif conservation, and a majority of NAC proteins (~89.9%, 62 of 69) had seven motifs, corresponding to five NAC DNA-binding subdomains, and two of the remaining seven proteins lacked only an E subdomain.

Subsequently, each NAC domain was classified, which revealed the E values of the A–D subdomains (E subdomain was not detected). The presence or absence of the various subdomains in each subfamily is listed in S4 Table. The results were consistent with those presented in S1 Fig, members of subgroups I–V generally contained a complete NAC domain, whereas those in subgroups VI–XIV contained an incomplete domain.

Comparative analysis of the NAC proteins from *Brachypodium* and *Arabidopsis*, which were the monocot and dicot model plants respectively, was also performed. From S3 Table, the members of subgroups VII–XIIV did not show high similarity with those of ANAC family except for subgroup X. Phylogenetic tree based on Bayesian MCMC methods (S3 Fig) showed that the classifications of the *Brachypodium* NAC family was almost identical and applicable to the *Arabidopsis* NAC family, especially, subgroups I–VI. Specifically, the subgroups VII–IX did not contain any ANAC members, and similarly ANAC–1 and -2 clustered ANACs alone, which coincided with that predicted previously (S3 Table, S3 Fig). Anyway, members in the subgroups shared the similar motif compositions likewise (S4 Fig). The type, order, and number of motifs were summarized in S5 Fig and similar to those in the *Brachypodium*. Statistical analysis revealed that the proportions of each subgroup were similar between *Brachypodium* and *Arabidopsis*, with only a small disparity in the subgroup III (10.2% in *Brachypodium* and 22.6% in *Arabidopsis*).

**Fig 3:**
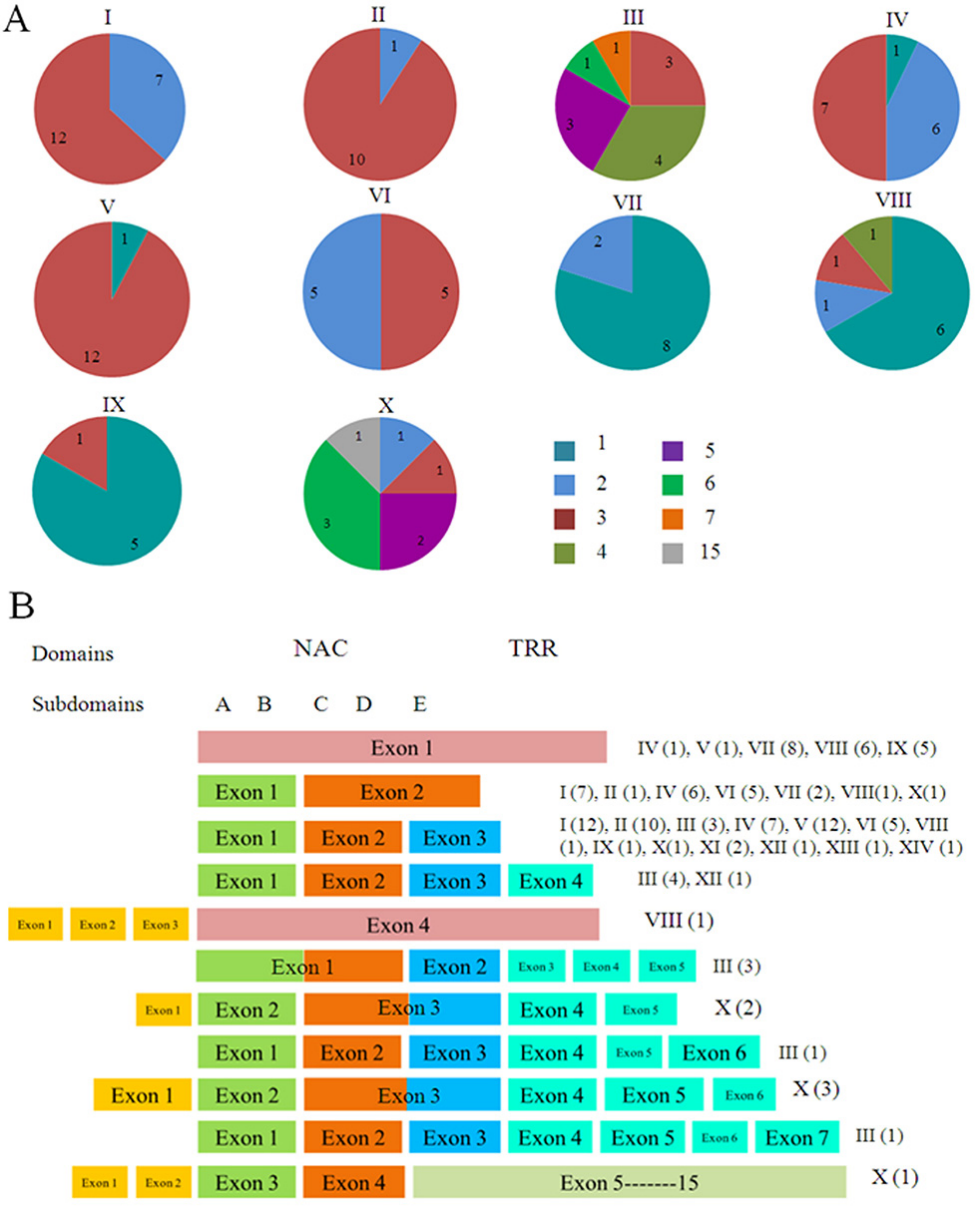
Schematic diagram of exon distribution of *Brachypodium* NAC genes. (A) Proportions of exon numbers within each subfamily. (B) The relationship of the substructures of BNAC proteins and the corresponding to exons.

Analysis of the exon/intron structures of the *BNAC* genes revealed that the number of exons differed among members of the BNAC gene family, mostly ranging from one to three, whereas *BNAC028* had the greatest number of exons, (up to 15 exons, S6 Fig). However, three of five subgroups in the Clade I showed significant proportion of three exons, except for the subgroup III (the number of exons ranged from two to seven) and subgroup IV (two exons accounted for 42.9% and three exons accounted for 50%), and the number of NAC members of the Clade I possessing three exons accounted for 63.8%. In contrast, the number of exons in the other subgroups varied greatly. Generally, 80%, 66.7% and 83.3% of the members of subgroups VII, VIII and IX had only one exon, respectively, whereas those in subgroup X had a greater number (Fig 3A). The similar results were obtained from the ANAC family genes (S4 Fig).

Furthermore, the relationship between the substructures of the protein and the corresponding exons was analyzed comprehensively (Fig 3B). Most NAC members from subgroups II and V and almost half of the members of the subgroups I and IV had three exons that aligned well, showing significantly similar structural features. Specifically, the first exon encodes the A and B subdomains, the second exon encodes C and D subdomains and the third exon begins with the E subdomain and contains all C-terminal regions including the TRR. Nevertheless, there were also several exceptions. The greatest variation was observed in subgroup X, which mainly occurred in the C-terminal region (Fig 3B).

### Functional divergence analysis of *Brachypodium* NAC proteins

The DIVERGE v3.0 software was used to identify the critical amino acid residues related to functional divergence using a framework based on Type-I functional divergence (site-specific shift of evolutionary rate) and Type-II functional divergence (site-specific shift of amino acid property) [39,40]. The methods are not sensitive to saturation of synonymous sites, therefore, they are applied extensively in research of various gene families [41,42]. The coefficient of Type-I functional divergence (θ_I_), which was statistically independent between the two gene clusters, was used to determine the Type-I of functional divergence [43]. As shown in Table 2, the θ_LTR_ value between subgroups II and III, III and VIII, III and IX was 3.642, 3.456 and 3.402 (range, 2.71–3.84), respectively, the coefficient of functional divergence (θ_I_) between them was statistically significant (*P* < 0.1). The coefficients of the five subgroup pairs (I/IV, I/X, II/X, IV/X and V/IX) were 0.488, 0.429, 0.415 0.746 and 1.209, respectively, which were moderately statistically significant (*P* < 0.05). The coefficients of the nine subgroup pairs (0.467–1.580) were highly statistically significant (*P* < 0.01). This suggests that specific sites might be retained selectively in certain members of the BNAC family, which led to evolution in the direction of subgroup-specific functions. Type-II functional divergence was divided into two cases: the coefficients of Type-II functional divergence (θ_II_) of some subgroup pairs were apparent, with values ranging from 0.031 to 0.662, whereas other pairs showed the opposite trend, in which the coefficients were not apparent, with values < 0 (Table 2). These results suggest that site-specific shifts of evolutionary rate and of amino acid property differed greatly among subgroup pairs and/or within each subgroup pair.

**Table 2:** Functional divergence between NAC subgroups in *Brachypodium distachyon*.

Subgroup 1	Subgroup 2	Type-I			Type-II	
		θ_I_ ± s.e.	LTR	Q_k_ > 0.9	θ_II_ ± s.e.	Q_k_ > 0.9
I	III	0.467 ± 0.172	8.164**	2	0.117 ± 0.477	1
I	IV	0.488 ± 0.205	4.649*	1	0.067 ± 0.426	2
I	V	0.473 ± 0.252	13.659**	17	0.137 ± 0.585	8
I	X	0.429 ± 0.232	3.946*	1	0.198 ± 0.433	8
I	VIII	1.182 ± 0.164	37.079**	24	0.662 ± 0.331	0
I	IX	0.502 ± 0.172	13.105**	3	0.367 ± 0.428	12
II	III	0.653 ± 0.251	3.642	1	0.068 ± 0.330	1
II	IV	0.549 ± 0.324	2.605	0	0.207 ± 0.252	6
II	V	0.128 ± 0.433	0.213	0	0.121 ± 0.475	6
II	X	0.415 ± 0.252	3.909*	0	0.327 ± 0.268	8
II	VIII	1.105 ± 0.251	15.871**	24	0.608 ± 0.265	0
II	IX	0.810 ± 0.287	12.122**	24	0.302 ± 0.319	8
II	VII	−0.816 ± 0.022	0	0	0.031 ± 0.656	2
III	X	−0.016 ± 0.378	0.013	0	−0.283 ± 0.454	1
III	VIII	0.594 ± 0.327	3.456	1	−0.541 ± 0.703	0
III	IX	1.371 ± 0.542	3.402	21	−0.943 ± 0.773	0
IV	X	0.746 ± 0.264	4.854*	1	0.166 ± 0.295	8
IV	VIII	1.245 ± 0.238	17.672**	24	0.090 ± 0.416	4
IV	IX	1.580 ± 0.243	16.928**	24	0.071 ± 0.369	0
IV	VII	0.066 ± 0.022	0	0	−0.070 ± 0.724	2
V	X	0.362 ± 0.207	2.448	0	0.280 ± 0.487	14
V	VIII	0.977 ± 0.300	9.734**	21	−0.187 ± 0.804	1
V	IX	1.209 ± 0.364	6.517*	19	−0.624 ± 0.877	0
X	VIII	0.084 ± 0.022	0	0	0.066 ± 0.505	6
X	IX	−0.189 ± 0.305	0.140	0	−0.122 ± 0.447	2
X	VII	−0.030 ± 0.022	0	0	0.306 ± 0.680	11

Note: θ_I_ and θ_II_, the coefficients of Type-I and Type-II functional divergence; LRT, Likelihood Ratio Statistic

*, p < 0.05

**, p < 0.01

Qk, posterior probability.

In addition, some critical amino acid residues that were responsible for functional divergence were predicted by setting suitable Qk values as a threshold. Here, Qk > 0.9 was set as the threshold to predict critical amino acid residues related to Type-I and Type-II functional divergence. Based on comparisons of Type-I and Type-II (S5 Table), the number and positions of the critical amino acid residues differed between each subgroup pair. Nevertheless, some critical sites (marked in bold font) were responsible for both Type-I and Type-II functional divergence, suggesting that they played important roles in functional divergence during the course of evolution.

### Analysis of promoter regions and miRNA-mediated regulation

Gene expression can be regulated by the binding of TFs to corresponding transcription factor binding sites (TFBSs) upstream of target genes. We analyzed the promoter sequences in 1500 bp region upstream of the start codon of *BNAC* genes (S6 Table). In a diverse range of metabolic activities, interactions of the *cis*-acting elements in promoter regions with various TFs regulate the expression of the downstream genes [44]. In particular, they play crucial roles in the developmental and/or environmental regulation of gene expression [45]. Of the seven types of regulatory elements, three were related to important physiological processes: light periods, hormonal/environment responses and developmental regulations. Several light-responsive elements were present in the promoter regions, including Box I [46], Box 4 [47], GAG-motif [48] and G-box [49]. Among these, the G-box element had more copies in the NAC family, particularly in the subgroup IV (a mean of 7.62). In addition, the sp1 also had a high abundance, ranging from 2.1 to 8.0 copies per subgroup (S6 Table). The results described above suggest that the *NAC* genes might be related to photosynthesis and/or carbohydrate metabolism. Phytohormones and other abiotic stress-responsive mechanisms also play a crucial role in plant self-defence against environmental stresses, such as ABRE [50], TGA-motif [51], TCA-motif [52] and MBS [53]. Of these, ABRE (72.2%, 35 of 118) is one of the most abundant hormone-related regulatory elements in *B*. *distachyon*, indicating that the expression of several *BNAC* genes is induced by ABA-mediated signal transduction. Obviously, the subgroup IV likely conferred a great advantage due to the high number of copies of each member (19.08 per gene). In addition, development-related elements are also present, including those related to meristem expression (CAT-box, CCGTCC-box and NON-box), circadian control (circadian element), endosperm expression (skn–1 motif and GCN4 motif) and other related regulations. Promoter analysis showed the presence of several *cis*-acting regulatory elements in the regions upstream of the *BNAC* genes, which further confirmed that *BNAC* genes are likely involved in regulating the growth, development and response to environmental stresses of plants.

Post-transcriptional regulation mechanisms mediated by microRNAs (miRNAs), ~22 nucleotide non-coding RNAs, are endemic in plants and animals, which regulated gene expression by targeting mRNAs for cleavage or translational repression [54,55]. We identified and analyzed miRNA164s and their corresponding targets in the NAC gene family in *B*. *distachyon* using the miRBase [56] and psRNATarget databases [57]. First, a total of 113 members of miR164 family were retrieved from 32 plant species in miRBase (version 20.0). Their sequences were collected into S7 Table. Among these, most shared an identical sequence (5′-UGGAGAAGCAGGGCACGUGCA–3′), which is regarded as the standard mature sequence of miR164, while other members showed 1–5 nucleotide differences compared with the standard sequence (Figure A in S2 File). In *Brachypodium*, five miRNA164s (Bdi-miRNA164a-c, e, f) were searched, and distributed on chromosomes 1, 2 and 3. Three members of the BNAC family (*BNAC012*, *BNAC078* and *BNAC108*) were targeted by miRNA164, which were divided into subgroup I (S2 File). The mature miRNAs were all 21 nucleotides in length, whereas the pre-miRNA sequences of *B*. *distachyon* were diverse both structurally and in terms of size, ranging from 127 to 209 bp (Figures B and C in S2 File and S8 Table).

### Membrane-bound BNAC subfamily

**Fig 4:**
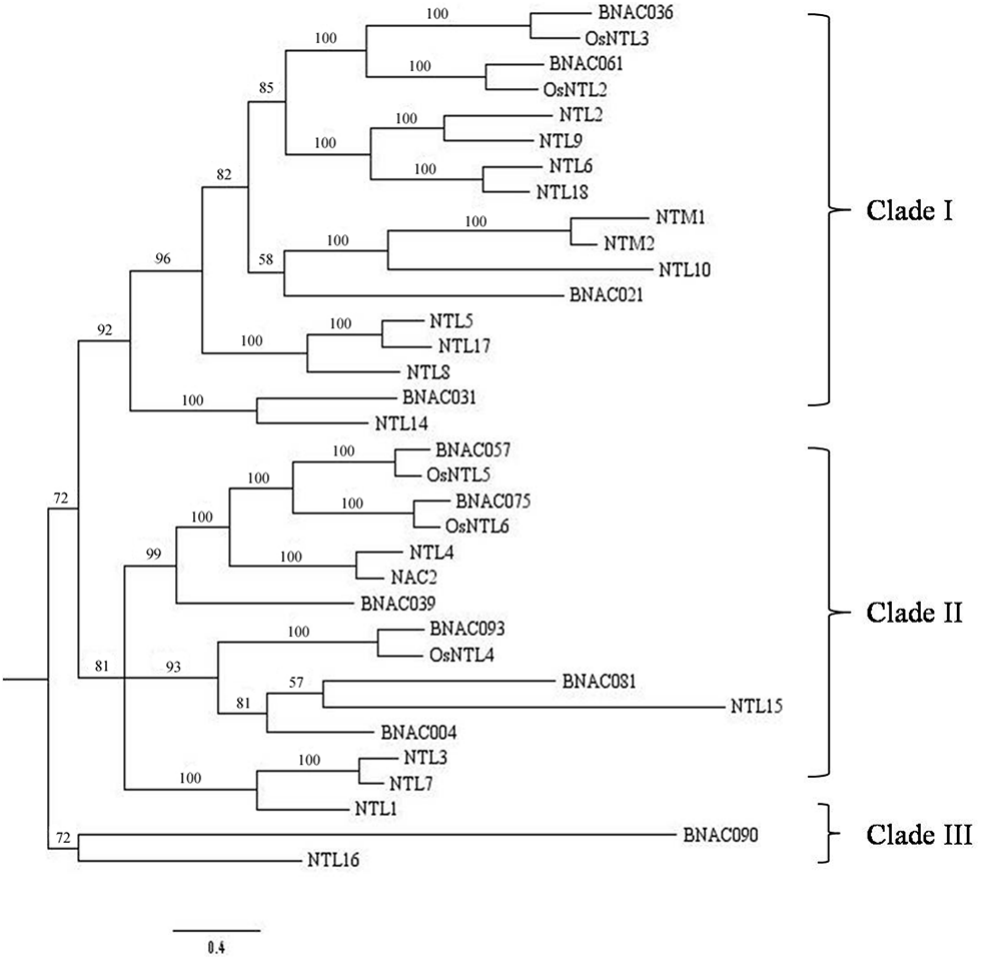
Compared analysis of phylogenetic relationships of NAC MTFs of *Brachypodium* with *Arabidopsis* NTL and OsNTL proteins. The phylogenetic tree was constructed using Bayesian MCMC algorithms. The Bayesian tree was divided into three clades.

With the aid of TMHMM Server v.2.0, 11 (~9.32%) BNAC proteins containing α-helical TMs were identified (Table 3), among which BNAC057 and BNAC075 were predicted to contain two TMs. Besides, the transmembrane motif in BNAC081 and BNAC069 is located at position of 7–25 and 7–29 of N-terminus, respectively. The redundant motifs and abnormal positions were also reported in other species. For example, soybean has 11 (~7.23%) predicted NTLs, and two proteins (GmNAC013 and GmNAC136) contain two TMs [16]. In addition, of the 17 (~8.33%) NTLs in Chinese cabbage,1 (Bra012470) contains a TM at its N-terminus [29]. A phylogenetic tree of membrane-bound NAC proteins from *Brachypodium*, *Arabidopsis* and rice was constructed based on Bayesian MCMC methods (Fig 4). Totals of 18 *Arabidopsis* MTFs (NTLs) and 5 rice MTFs (OsNTLs) were identified using TMHMM Server v.2.0 and Aramemnon (http://aramemnon.botanik.uni-koeln.de/) and were named based on previous reports [16,26]. As shown in Fig 4, the tree was divided into three clades. Of these, Clade I shared the greatest number (17) of NTLs, followed by Clade II (15), while most of BNAC were distributed in Clade II. They may be candidate genes for identifying functions of BNAC MTFs.

**Table 3:** Putative *Brachypodium* NAC membrane-bound transcription factor (NTLs).

Names	Locus name	Size (aa)	Transmembrane regions^a^
BNAC004	Bradi1g14461	302	220–246
BNAC021	Bradi1g52480	565	539–561
BNAC031	Bradi1g77217	710	687–708
BNAC036	Bradi2g09530	495	462–484
BNAC039	Bradi2g24790	464	416–436
BNAC057	Bradi3g12470	653	411–438 624–644
BNAC061	Bradi3g16480	800	777–799
BNAC075	Bradi3g56080	648	559–576 620–639
BNAC093	Bradi4g34022	689	652–670
BNAC081	Bradi4g13586	204	7–25
BNAC090	Bradi4g26470	573	7–29

a were identified using the TMHMM Server v.2.0.

### Expression profiles of *BNAC* genes under different abiotic stress conditions

**Fig 5:**
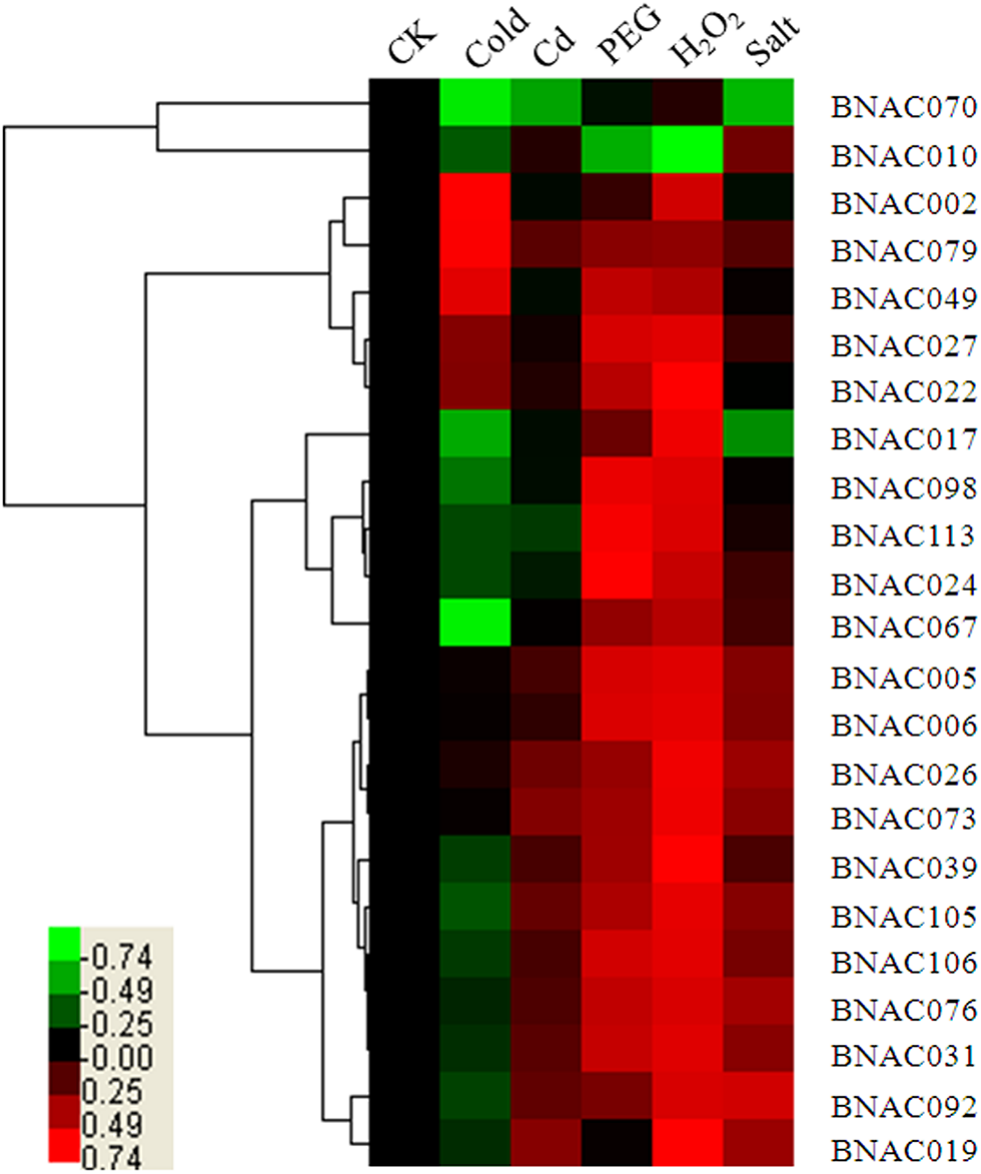
Expression profiles of *BNAC* genes in response to abiotic stresses. The heat map was generated using cluster 3.0 software. The relative expression values were log2 transformed. The bar at the bottom of the heat map represents the relative expression values.CK, before treatment; Cd, cadmium stress.

**Fig 6:**
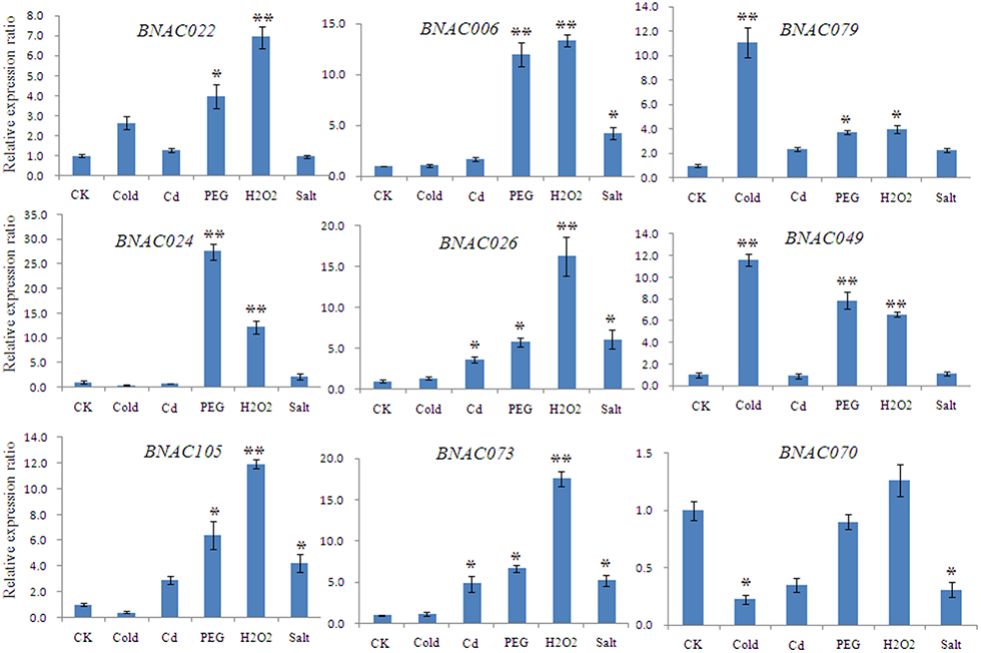
The relative expression ratio of 9 representative *BNAC* genes in different abiotic stresses. The single and double asterisks indicate genes whose expression was up- or down-regulated by more than three- and tenfold, respectively. The y-axis represents the relative expression level of the stresses-treated seedling compared with that of control seedling. CK, before treatment; Cd, cadmium stress. Error bars represent the standard errors.

To investigate the roles of *NAC* genes in *B*. *distachyon* under diverse environmental stresses, a total of 23 *BNAC* genes were selected and their expression patterns were analyzed quantitatively in response to the following five abiotic stresses: cold, cadmium, drought, H_2_O_2_ and salt. A heat map representation of expression in response to the five stresses was shown in Fig 5. From the heat map, all the analyzed *BNAC* genes displayed variations in their expression quantity in response to one or more stresses, of which the majority of *BNAC* genes were regulated under drought, H_2_O_2_ and salt conditions, and approximately half were regulated under cold and cadmium conditions. Of these, the expression levels of 18, 17 and 11 genes were up-regulated more than threefold under drought, H_2_O_2_ and salt stresses, respectively. Only four and two genes were up-regulated more than threefold by cold and cadmium stress, respectively. During the five stress treatments, the following eight genes accounted for 34.8% of the significant changes in expression (> 10-fold up-regulation) in one or two stresses: *BNAC006*, *BNAC022*, *BNAC024*, *BNAC026*, *BNAC049*, *BNAC073*, *BNAC079* and *BNA105*, among which five were divided into the SNAC subfamily. Only *BNAC070* was induced by only one stress (H_2_O_2_, Fig 6).

**Fig 7:**
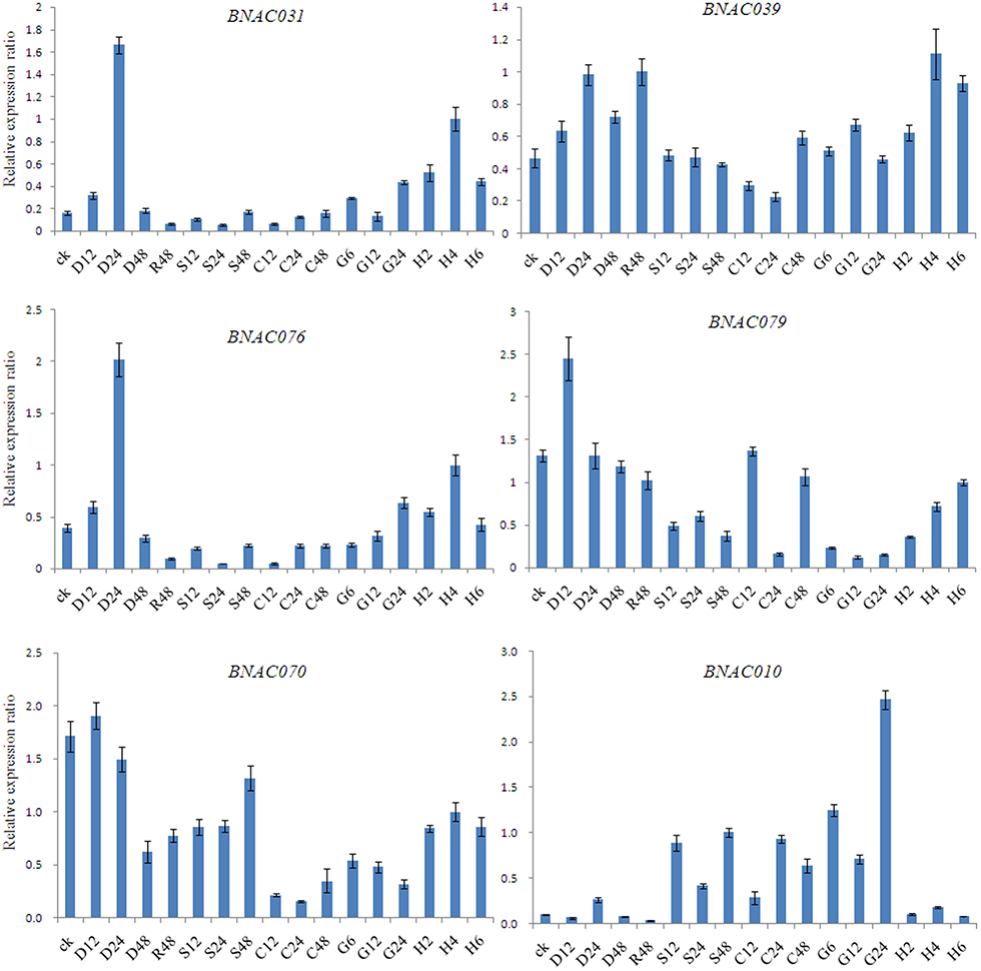
The relative expression ratio of 6 representative *BNAC* genes in response to different abiotic conditions. The name of each gene is provided at the top of each bar diagram (error bars indicate standard deviations). D12, D24, D48 and R48: drought treatments for 12, 24, 48 h and recover 48 h, respectively; S12, S24 and S48: salt treatment for 12, 24 and 48 h, respectively; C12, C24 and C48: cold treatment for 12, 24 and 48 h, respectively; G6, G12 and G24: gibberellin treatment for 6, 12 and 24 h, respectively; H2, H4 and H6: H_2_O_2_ treatment for 2, 4 and 6 h, respectively.

To further investigate the dynamic changes in gene expression, six representative genes were analyzed using qRT-PCR (Fig 7). During drought treatment from 200 mM PEG6000, four genes were up-regulated at 24 h, of which *BNAC031* and *BNAC076* were induced significantly. Whereas the expression of *BNAC070* and *BNAC079* was down-regulated significantly from 12 to 48 h. Further analyses showed that the expression of only *BNAC039* and *BNAC070* increased after recovery compared with that at 48 h under drought treatment, suggesting that both genes are sensitive to environmental stress. The expression of four genes (*BNAC010*, *BNAC031*, *BNAC070* and *BNAC079*) was down-regulated at 24 h compared to that at 12 h under salt treatment. However, the expression of *BNAC079* was up-regulated at 24 h. Under cold treatment, the expression of most of genes showed subtle variations from 12 to 48 h, with the exception of *BNAC079*, which was down-regulated significantly at 24 h. Obviously, five of the six genes were expressed at the highest levels at 4 h under H_2_O_2_ stress. As shown in Fig 7, the expression of five genes (except for *BNAC070*) was not a single trend by treatment with gibberellin. Furthermore, the expression of several genes was distinct after 6, 12 and 24 h: there were small changes in *BNAC079* expression, whereas that of *BNAC010*varied greatly during the period of stress exposure.

## Discussion

### Evolutionary variation and duplication patterns of *Brachypodium NAC* gene family

Molecular characterizations revealed great variations, of which the motif compositions differed markedly among the subfamilies, whereas the NAC proteins clustered in the same subfamily shared a similar motif composition (S1 Fig). Such motif variation specifies the diversification in biological functions [11]. Obviously, the N-terminal regions exhibited conserved motif compositions, whereas the C-terminal regions shared variant regions, indicating that the major differences in the NAC sequences among various subfamilies were present mainly in the C-terminal TRR, which is consistent with the viewpoint that C-terminal regions are highly divergent [14]. Nevertheless, the divergent C-terminal regions of NAC proteins generally operate as functional domains (activators or repressors), which might explain the divergent functions of these proteins. In addition, ANAC family and BNAC family existed variant motif compositions in the Clade II, indicating that they may result from independent evolutionary events.

Gene duplications have been one of the primary driving forces in the evolution of genomic and genetic systems [58], and are a major mechanism for the establishment of new functions [59]. A recent report revealed that 70%–80% of angiosperms have undergone duplication events [60]. In the current study, the time of segmental duplication ranged from 45.38 to 75.08 MYA. However, the divergence time of *Brachypodium* from wheat was 32–39 MYA, and that from rice and sorghum was 40–60 MYA [30], indicating that the duplication of *Brachypodium* NAC gene family occurred before the divergence from wheat, and almost concurrently withits divergence of genome from rice and sorghum. Furthermore, the genomic duplication of *Brachypodium* occurred 56–72 MYA, which is consistent with approximately half of these genes and the rest genes occurred duplication during the diversification of the grasses.

### Phylogenetics and functional divergence of *NAC* gene family

NAC transcription factors have been annotated functionally in other higher plants, such as *Arabidopsis*, rice and soybean. Proteins with similar domains may have the same or similar biological functions [61]. Such phylogeny-based function prediction has been applied to other species [13,62]. Therefore, the phylogenetic analysis of *Brachypodium* NAC family and published ANAC proteins with known functions contributes to predicting the possible functions of *BNAC* genes belonging to the same subfamily. The known NAC members are involved in diverse aspects of plant growth and development, and stress responses, revealing that the high sequence diversification of the NAC family induces functional diversity [13]. Together, the information described above provides a solid basis for identifying the *Brachypodium* NAC family genes. Up to date, three phylogenetic analyses of NAC proteins have been published based on NAC domain (S9 Table). The first study was a comprehensive analysis of the NAC family in 105*Arabidopsis* and 75 rice sequences based on subdomains A–E, which were divided into two groups (I and II) and 18 subfamilies [12]. Nuruzzaman et al. [25] classified the OsNAC gene family into two major groups (A and B) and 16 subgroups: Group A did not include any of analyzed members of NAC, consistent with the current analysis. Shen et al [63] divided the NAC family into eight subfamilies and summarized the known functions of the NAC-a, NAC-b, NAC-c and NAC-d subfamilies: (a) responses to biotic or abiotic stresses, (b) cytokines with roles in signaling during cell division or endoplasmic reticulum stress responses, (c) regulation of plant cell wall development, and (d) organ initiation and formation, respectively. This is consistent with our phylogenetic classifications. This comparative analysis is beneficial for a more comprehensive understanding of the *BNAC* genes.

During gene duplication, significant site-specific changes may result in selective functional constraints between gene clusters of a family, leading to subgroup-specific functional evolution after diversification [64]. Generally, an amino acid residue is highly conserved in one duplicate gene, but highly variable in the other [65]. The accumulation of amino acid site mutations could lead to functional divergence of duplicated genes [60]. Therefore, the contributors between nine subfamilies to functional divergence can be determined by detecting critical amino acid sites (S5 Table). Furthermore, determining the suitable cut-off values is also essential. When Qk value < 0.9, there will be too many residues being fallen into contributors to functional divergence, while Qk value > 0.95 may ignore certain pivotal sites. Hence, we used Qk value > 0.9 in this study to reduce false positives.

### Expression and regulation of *NAC* genes

In this study, using qRT-PCR combined with the sequence similarity comparisons and phylogenetic analyses, we identified 23 genes that treated with five abiotic stresses (cold, cadmium, drought, H_2_O_2_ and salt). The different expression patterns (Fig 5) provide crucial information for determining gene functions [9], and indicate that these genes are involved in several signal transduction pathways [27]. Surprisingly, the majority of genes were up-regulated slightly and some (*BNAC024*, *BNAC070* and *BNAC0113*) were down-regulated in response to cadmium, manifesting that these genes cannot respond rapidly to such a short duration of stress (3 h). Obviously, all seven *BNAC* genes divided into the subgroup IV showed stress responses, consistent with previous reports [27,29], suggesting that this subgroup is stress-responsive. Subfamily IV contains the ATAF subfamily and the NAP subfamily (S9 Table). *ATAF1* and *RD26* are involved in the ABA pathway [66], while *NAP* regulates stress-resistant processes via both ABA-dependent and ABA-independent pathways [67], indicating that *NAC* genes belonging to subfamily IV are involved in ABA-dependent and -independent pathways. It is noteworthy that most of the selected genes are up-regulated under drought, salt and H_2_O_2_ conditions, but down-regulated under cold conditions (Fig 5). It is known that osmotic stresses caused by drought and salt participate in the ABA signaling pathway by activating SnRK2s, which subsequently activates AREB/ABF transcription factors through multiple-site phosphorylation, to regulate ABRE-dependent gene expression [68]. However, the responses to cold stress are mediated via ABA-independent pathway, among which it tempts CDPK up-regulation by destroying calcium ion balance, increasing the expression of ABF TF. Furthermore, some genes, such as *BNAC010* and *BNAC017*, exhibited opposing expression patterns under different stress conditions, which indicate that these are involved in the communication between different signal transduction pathways. In summary, NAC genes are regulated by both ABA-dependent and ABA-independent pathways on account of various promoter elements [9], while the expression of *BNAC* genes is likely to be regulated mainly through ABA-dependent pathways.

Interestingly, *BNAC002* was orthologous of *ATAF2* (*At5g08790*) and up-regulated under the stresses of cold and H_2_O_2_, while *ATAF2* was known as repressor of pathogenesis-related genes [69]. *NST1* (*At2g46770*), as a key regulator of the formation of secondary walls woody tissues [70], showed higher similarity with *BNAC019*, while *BNAC019* was down-regulated by cold stress and showed high expression in H_2_O_2_ and Salt. The genes with higher structural similarity may be conserved in the functions among species, which contributed to identifying the potential functions of such *BNAC* genes.

In addition, the temporal expression of each gene varied in response to the five stresses (Fig 7). Plant hormones play pivotal roles in regulating various plant processes―such as signaling and gene expression―during abiotic and biotic stresses [15]. They influence signaling responses by acting in conjunction with or antagonizing each other to maintain the cellular homeostasis [66]. Together, the results described above suggest that certain *BNAC* genes show stress-specific and/or time-specific responses.

In fact, gene expression is regulated by multiple levels of control. Proper control of the expression level and the activity of target genes are essential [8]. At transcriptional level, gene expression is regulated by the binding of specific TFs to its regulatory region [9]. The sites recognized by numerous TFs are present in *NAC* promoters (S5 Table), including Skin–1 motif, GCN4_motif, CCGTCC-box and RY-element (development-related elements), as well as ABRE, DRE, MBS and W-box (hormonal/environment response-related elements), supporting that NAC TFs are involved in plant developmental programs and stress responses. It is well recognized that the miRNA-mediated cleavage of genes is important for post-transcriptional regulation. Most predicted targets of miRNAs are transcription factors, which play roles in developmental timing, patterning or cell differentiation [71,72]. MiRNA164 targets NAC domain-encoding mRNAs (such as *CUC1/2*, *NAC1*), which is necessary for lateral organ enlargement, floral development and responses to abiotic stress [73–77]. Therefore, the predicted miRNA164-targeted genes (*BNAC012*, *BNAC078* and *BNAC108*) in this study likely have functions identical to those of the *CUC*1/2 genes.

**Fig 8:**
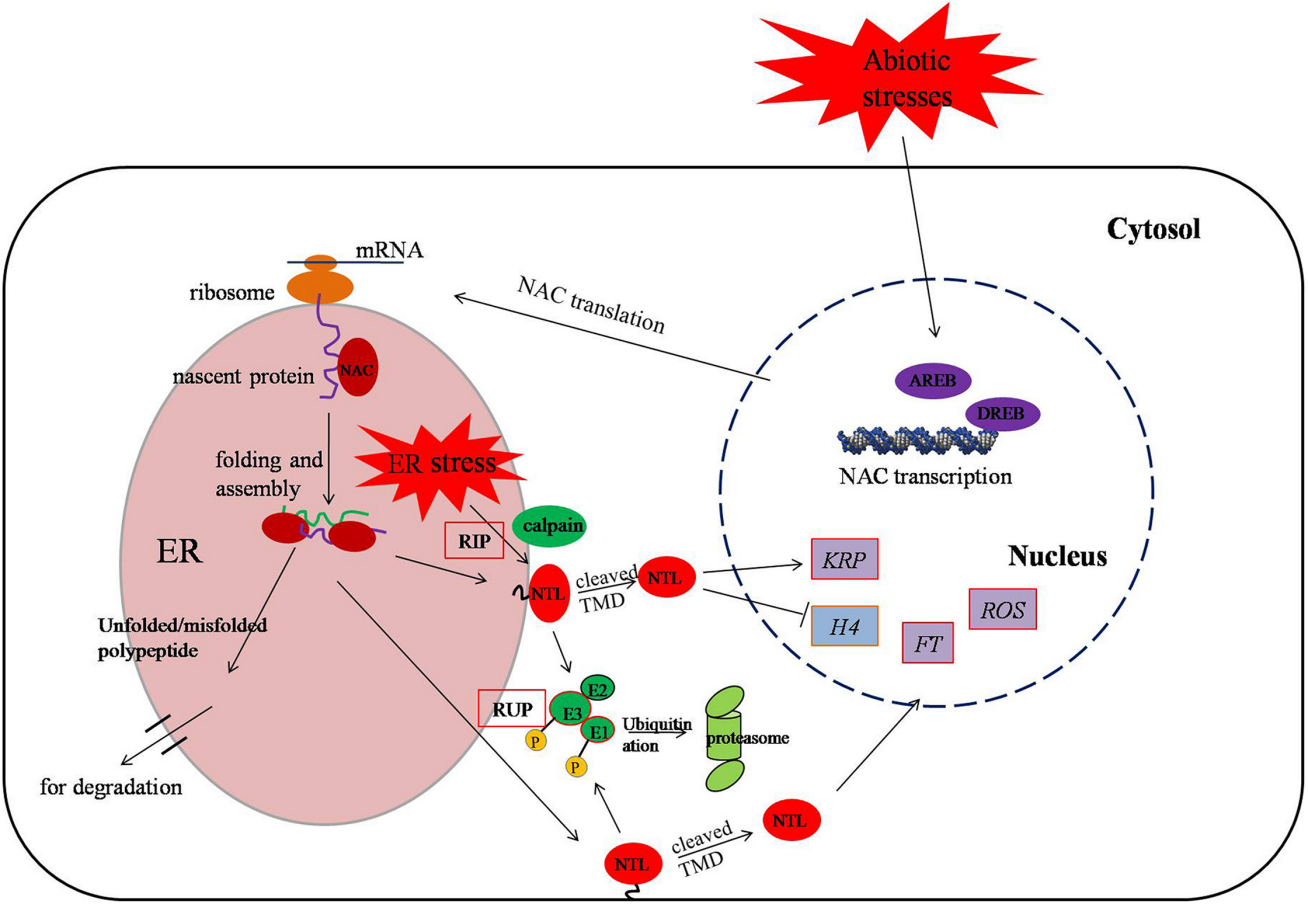
A putative pathway of membrane-bound NAC TFs in response to various abiotic stresses. Transcription factors such as DREBs or AREBs might regulate the transcription of *NAC* genes by binding to stress-related *cis*-acting elements in the upstream promoter. The NTLs are released from RIP and RUP in response to ER stress. During RIP, activated NTLs are released from membrane by specific membrane-integrated proteases, such as calpain. During RUP, the NTLs are ubiquitinated and degraded by the 26S proteasome to maintain the protein stability. DREB, dehydration responsive element binding protein; AREB, ABA-responsive element binding protein; ER, endoplasmic reticulum; RIP, regulated intramembrane proteolysis; RUP, regulated ubiquitin/proteasome-dependent processing; NTL, NAC membrane-bound TF; KRP, KIP-related protein; H4: histone H4; FT, FLOWERING LOCUS T; ROS, reactive oxygen species.

Furthermore, NAC proteins with a TM are involved in post-translational regulations via two mechanisms: regulated intra-membrane proteolysis (RIP) and regulated ubiquitin/proteasome-dependent processing (RUP) (Fig 8) [78]. Under abiotic stresses, the gathering NTLs unfolded or misfolded is released from the membranes in response to ER stress. During RIP, specific membrane-integrated proteases, including calpain, cleave the transmembrane domain (TMD) of NTLs. The resulting activated NTLs are transferred into the nucleus, in which they target the corresponding genes. On the other hand, the protein stability of activated NTLs is modulated further by repression of the ubiquitin or 26S proteasome pathway. Ubiquitination not only degrades unfolded and misfolded proteins but also attenuates auxin signaling for proteasomal degradation [9]. In our analysis, BNAC031 and BNAC039 with a TM were up-regulated in treatment of PEG, H_2_O_2_ and Salt, verifying that the kind of proteins respond to abiotic stresses. Admittedly, the activated NTM1 regulates cell division by inducing a subset of CDK inhibitor genes (*KRPs*) and suppressing histone *H4* expression [79]. Furthermore, NTL4 and NTL9 induce leaf senescence by mediating osmotic stress signaling [19,80]. Salt-mediated NTL8 delays flowering by repressing *FT* expression, whereas GA-mediated salt signaling may regulate seed germination [18,81]. It is evident that NTLs have distinct or overlapping functions during stress responses [78]. Hence, comparison analysis of *Brachypodium* NTLs with *Arabidopsis* and rice NTLs (Fig 4) contributes to validating the putative functions in the future works.

## Conclusions

The current analyses demonstrated that segmental and tandem duplications may be responsible for the expansion of the *Brachypodium* NAC gene family. Motif compositions and exon-intron organizations analyses of BNACs and ANACs revealed their similar NAC architecture. Type-I and Type-II functional divergence might be relevant to functional classification of NAC gene family. The predicted functions of some *BNAC* genes were consistent with the phylogeny-based functional prediction. qRT-PCR revealed that *BNAC* genes were regulated under various abiotic stresses, which may manifest their involvement in general stresses response rather than stress-specific responses. These results not only provide a better understanding of the structures and functions of BNAC genes but will also facilitate genome-wide studies of NAC transcription factors in several important related species.

## Supporting Information

S1 Fig Conserved motifs of BNAC proteins. The schematic diagram of motifs was derived from MEME program. Each motif is represented by a colored box, whose order was automatically generated according to scores shown at the bottom. A detailed motif introduction is shown in S2 Fig.
(PDF)

S2 Fig Schematic diagram of NAC protein motifs in *Brachypodium*. The schematic diagram was derived from MEME. The order of motifs in the diagram was automatically generated by MEME according to scores.
(PDF)

S3 Fig Comparative analysis of phylogenetic tree of NAC members from *Brachypodium* and *Arabidopsis*. The amino acid sequences of 118 *BNAC* and 115 *ANAC* genes were aligned using MUSCLE program and the trees were generated based on Bayesian inference using Markov Chain Monte Carlo (MCMC) methods.
(PDF)

S4 Fig Conserved motifs and exon-intron organizations of *Arabidopsis* NAC genes. The methods generating the diagram were based on those used in BNAC genes. A detailed motif introduction is shown in S5 Fig.
(PDF)

S5 Fig Schematic diagram of NAC protein motifs in *Arabidopsis*. The schematic diagram was derived from MEME. The order of motifs in the diagram was automatically generated by MEME according to scores.
(PDF)

S6 Fig Exon-intron structures of *B*. *distachyon* NAC genes. The green boxes represent exons, the black solid lines connecting two neighboring exons represent introns and the blue boxes represent 5’-UTR and 3’-UTR. The grid scales show the gene sizes (kb).
(PDF)

S1 File Double standard curve and dissolution curve. Of 23 *BNAC* genes under different abiotic stresses **(Figure A)**. Of 6 representative *BNAC* genes during drought and salt stresses **(Figure B)**. Of 6 representative *BNAC* genes during cold, gibberellin and H_2_O_2_ stresses **(Figure C)**. The red standard curve represents reference genes and other blue curve represents *BNAC* genes.
(PDF)

S2 File Mature miR164 sequence analysis in plants and illustration of *BNAC* mRNA cleavage by Bdi-miR164. Sequence LOGO view from 113 mature miRNA164 sequences. The height of the letter at each position represents the degree of conservation **(Figure A)**. The predicted structure of the Bdi-miR164 synthetic precursors **(Figure B)**. Mapping of *BNAC* mRNA cleavage sites. The red box represents target sequences of mature miR164. The cleavage sites are indicated by arrows **(Figure C)**.
(PDF)

S1 Table Primer sequences used for quantitative real-time PCR. (DOCX)

S2 Table Lists of NAC protein homologs among *Brachypodium*, *Arabidopsis* and rice. (XLSX)

S3 Table Characteristics of the 118 *BNAC* genes and their deduced proteins. (XLSX)

S4 Table Identifications the presence or absence of *Brachypodium* NAC subdomains. (XLSX)

S5 Table Critical amino acid sites of functional divergence between subgroups of the NAC family in *B*. *distachyon*. (DOCX)

S6 Table Functions and number of identified *cis*-regulatory elements of *Brachypodium distachyon* NAC genes. (XLSX)

S7 Table Mature miRNA164 sequences of 113 members from 32 plant species. (TXT)

S8 Table List of mature miRNA164s and targeted NAC genes in *Brachypodium distachyon*. (XLSX)

S9 Table Comparisons of phylogenetic classifications of *BNAC* genes. (PDF)

## Abbreviations

ATAF: Arabidopsis transcription activation factor
BLAST: Basic local alignment search tool
CUC: Cup-shaped cotyledon (CUC), a NAC protein
MCMC: Markov Chain Monte Carlo
miRNA: microRNA, small regulatory RNAs
MTF: membrane-bound transcription factors
NAC: NAM, ATAF1,2, CUC2, a plant-specific TF family
NAM: No apical meristem (NAM), a NAC protein
NCBI: National Center for Biotechnology Information
NTL: NTM1-like, a membrane-associated NAC protein
RIP: regulated intramembrane proteolysis
RUP: regulated ubiquitin/proteasome-dependent processing
qRT-PCR: quantitative real-time polymerase chain reaction
SNAC: Stress-related NAC
TERN: Tobacco elicitor-responsive NAC
TFBS: Transcription factor binding sites
TIP: TCV-interacting protein
TM: transmembrane motif
HMM: Hidden Markov Mode
TRRs: transcription regulatory regions
VND: vascular-related NAC-domain
