# Supplementary material for: Molecular Characterization and Expression Profiling of NAC Transcription Factors in Brachypodium distachyon L
Source: PLoS One. 2015 Oct 7;10(10):e0139794. doi: 10.1371/journal.pone.0139794 (PMC4596864; doi:10.1371/journal.pone.0139794)
Supplement: S1 Fig — The schematic diagram of motifs was derived from MEME program. Each motif is represented by a colored box, whose order was automatically generated according to scores shown at the bottom. A detailed motif introduction is shown in S2 Fig. (PDF) [file pone.0139794.s001.pdf]

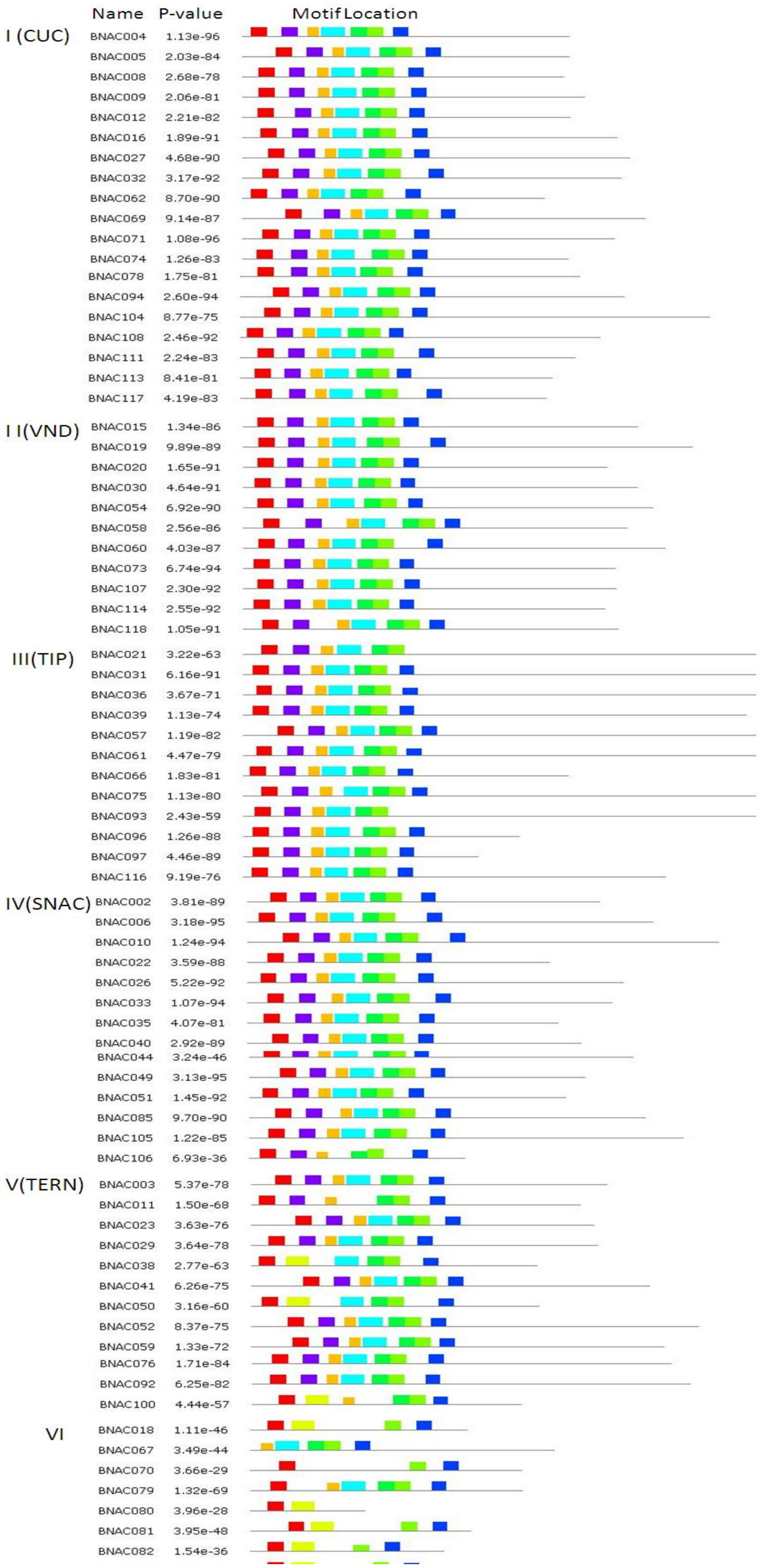
Name P-value Motif Location
I (CUC)
BNAC004 1.13e-96
BNAC005 2.03e-84
BNAC008 2.68e-78
BNAC009 2.06e-81
BNAC012 2.21e-82
BNAC016 1.89e-91
BNAC027 4.68e-90
BNAC032 3.17e-92
BNAC062 8.70e-90
BNAC069 9.14e-87
BNAC071 1.08e-96
BNAC074 1.26e-83
BNAC078 1.75e-81
BNAC094 2.60e-94
BNAC104 8.77e-75
BNAC108 2.46e-92
BNAC111 2.24e-83
BNAC113 8.41e-81
BNAC117 4.19e-83
I I(VND)
BNAC015 1.34e-86
BNAC019 9.89e-89
BNAC020 1.65e-91
BNAC030 4.64e-91
BNAC054 6.92e-90
BNAC058 2.56e-86
BNAC060 4.03e-87
BNAC073 6.74e-94
BNAC107 2.30e-92
BNAC114 2.55e-92
BNAC118 1.05e-91
III(TIP)
BNAC021 3.22e-63
BNAC031 6.16e-91
BNAC036 3.67e-71
BNAC039 1.13e-74
BNAC057 1.19e-82
BNAC061 4.47e-79
BNAC066 1.83e-81
BNAC075 1.13e-80
BNAC093 2.43e-59
BNAC096 1.26e-88
BNAC097 4.46e-89
BNAC116 9.19e-76
IV(SNAC)
BNAC002 3.81e-89
BNAC006 3.18e-95
BNAC010 1.24e-94
BNAC022 3.59e-88
BNAC026 5.22e-92
BNAC033 1.07e-94
BNAC035 4.07e-81
BNAC040 2.92e-89
BNAC044 3.24e-46
BNAC049 3.13e-95
BNAC051 1.45e-92
BNAC085 9.70e-90
BNAC105 1.22e-85
BNAC106 6.93e-36
V(TERN)
BNAC003 5.37e-78
BNAC011 1.50e-68
BNAC023 3.63e-76
BNAC029 3.64e-78
BNAC038 2.77e-63
BNAC041 6.26e-75
BNAC050 3.16e-60
BNAC052 8.37e-75
BNAC059 1.33e-72
BNAC076 1.71e-84
BNAC092 6.25e-82
BNAC100 4.44e-57
VI
BNAC018 1.11e-46
BNAC067 3.49e-44
BNAC070 3.66e-29
BNAC079 1.32e-69
BNAC080 3.96e-28
BNAC081 3.95e-48
BNAC082 1.54e-36

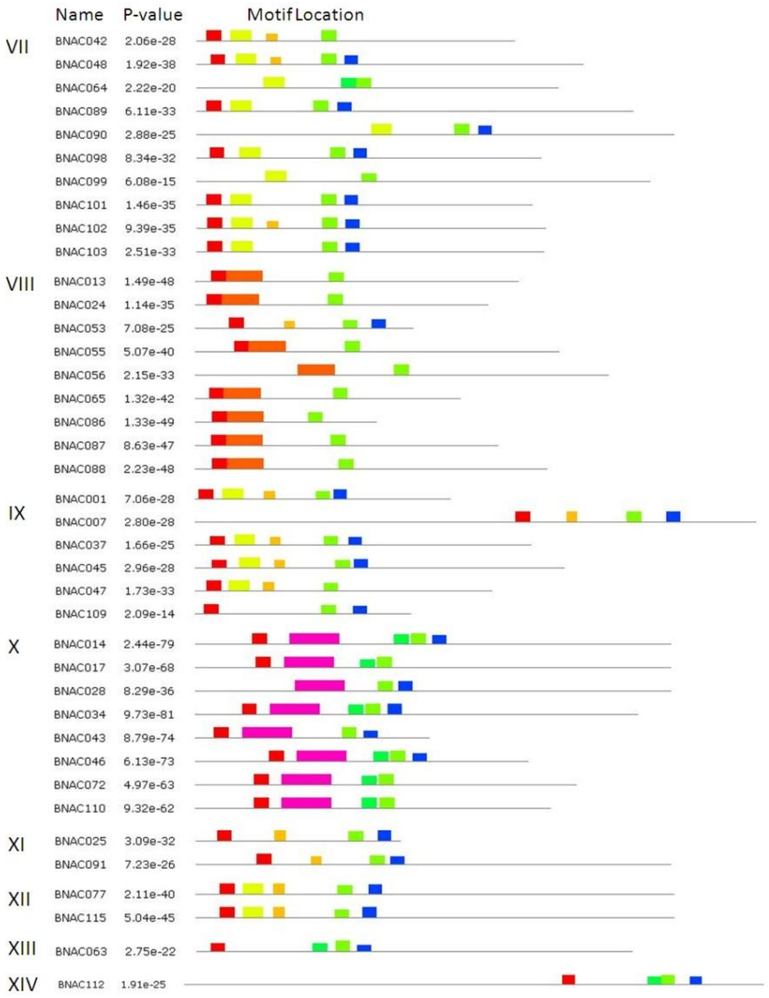

S1 Fig. Conserved motifs of BNAC proteins. The schematic diagram of motifs was derived from MEME program. Each motif is represented by a colored box, whose order was automatically generated according to scores shown at the bottom. A detailed motif introduction is shown in S2 Fig.
