# Supplementary material for: Molecular Characterization and Expression Profiling of NAC Transcription Factors in Brachypodium distachyon L
Source: PLoS One. 2015 Oct 7;10(10):e0139794. doi: 10.1371/journal.pone.0139794 (PMC4596864; doi:10.1371/journal.pone.0139794)
Supplement: S2 Fig — The schematic diagram was derived from MEME. The order of motifs in the diagram was automatically generated by MEME according to scores. (PDF) [file pone.0139794.s002.pdf]

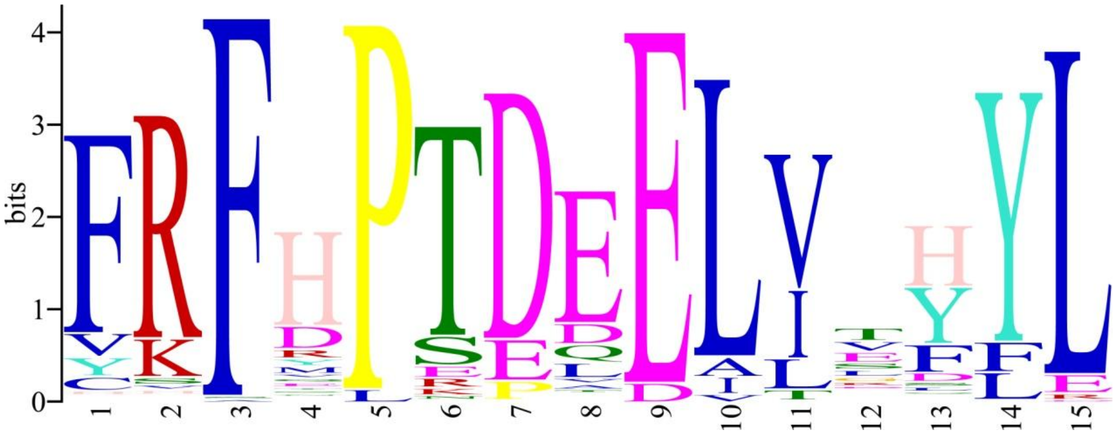

A subdomain

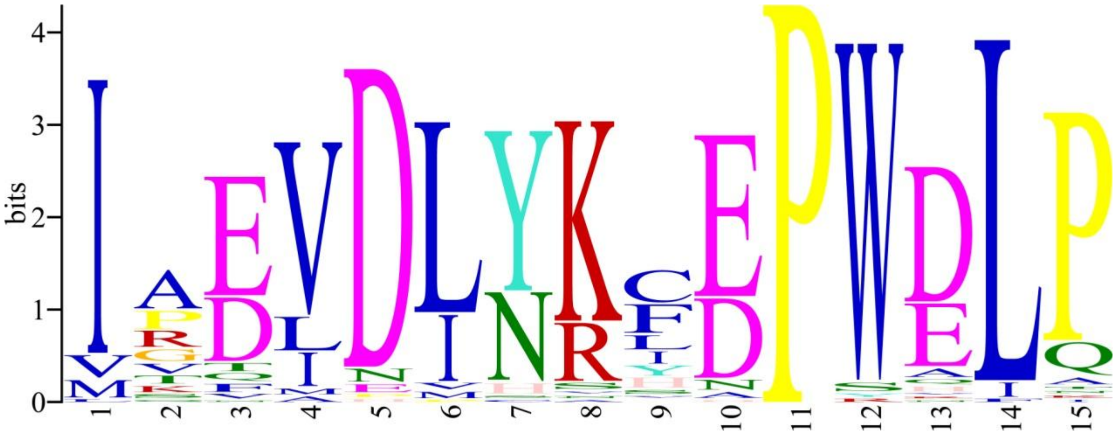

B subdomain

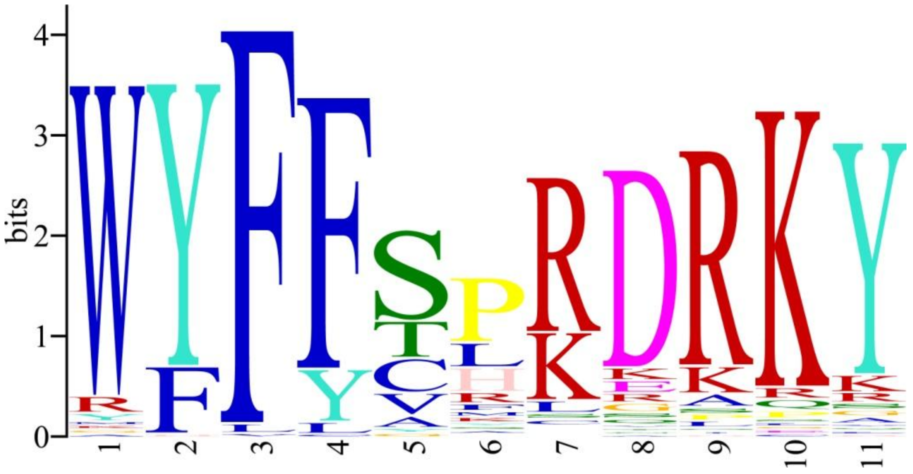

C1 subdomain

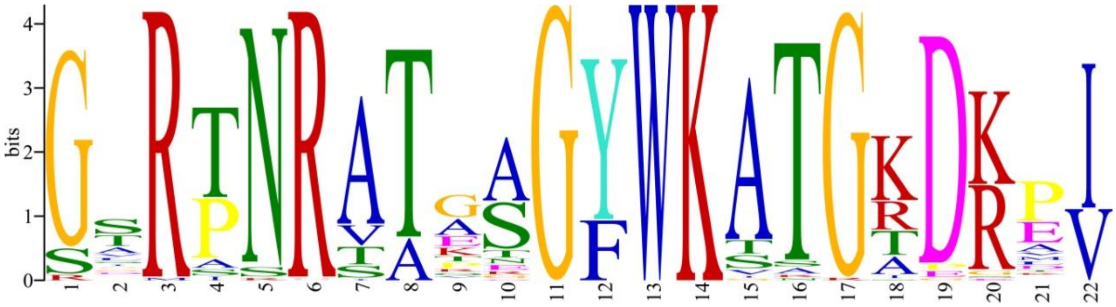

C2 subdomain

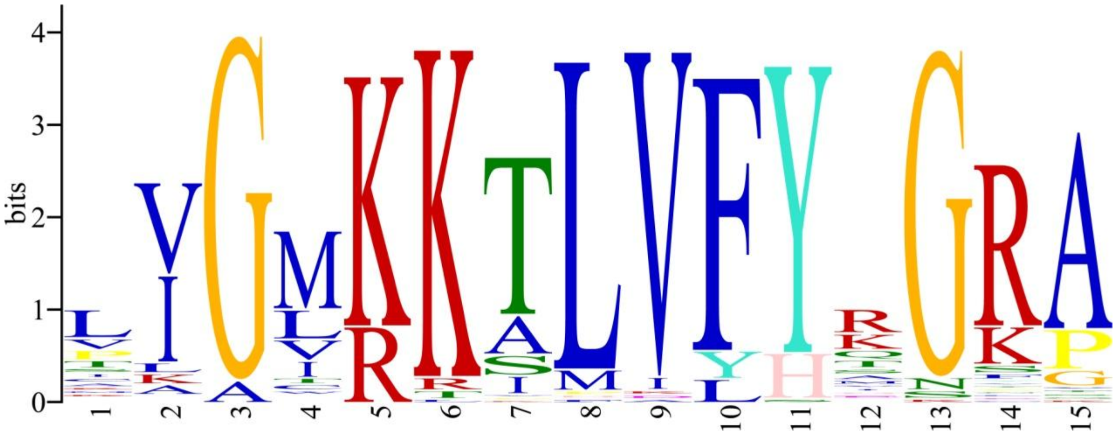

D1 subdomain

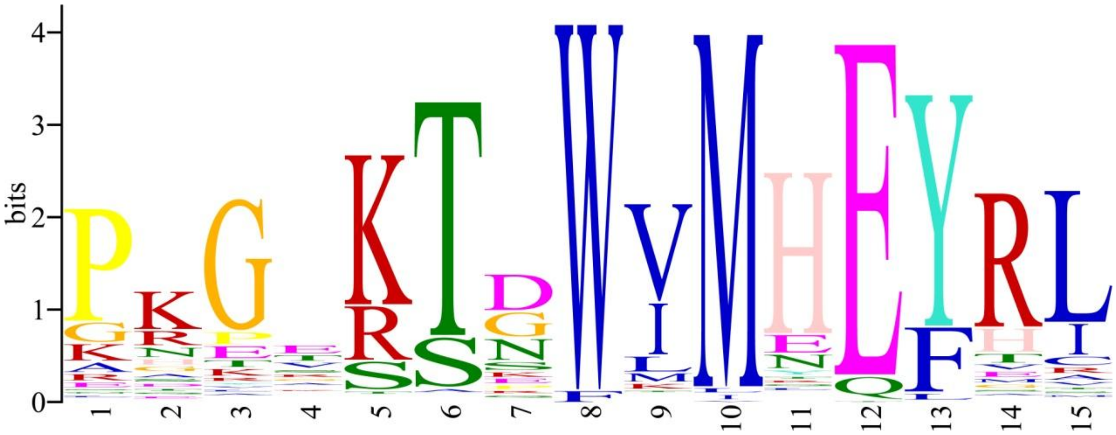

D2 subdomain

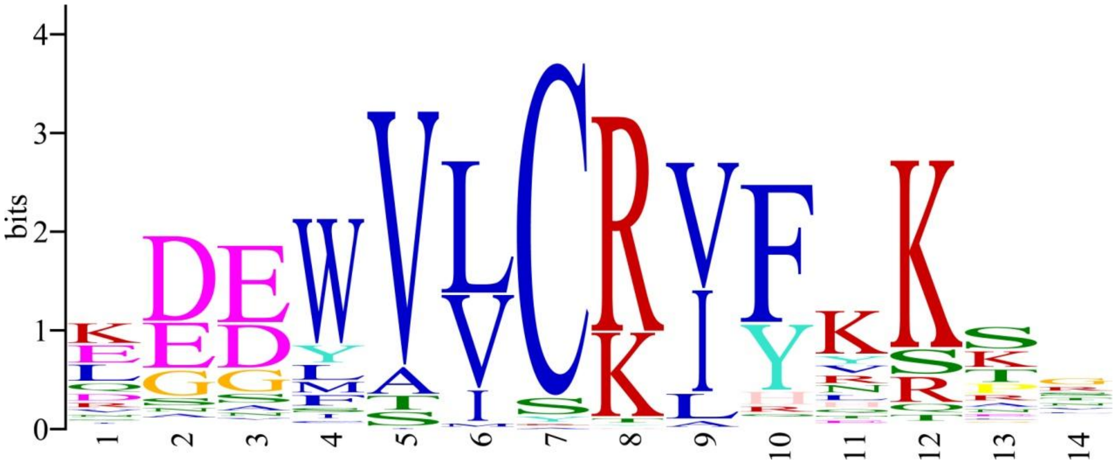

E subdomain

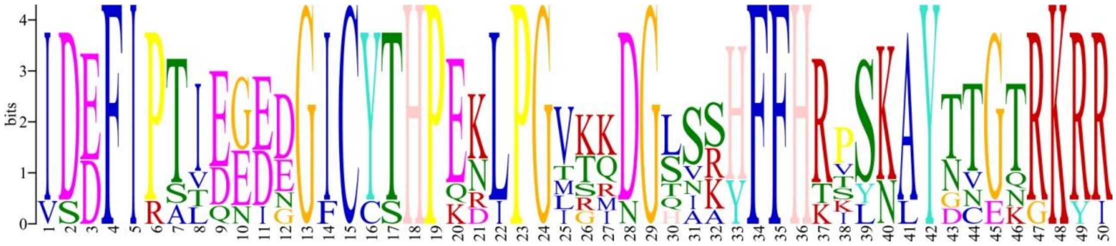

Motif 8

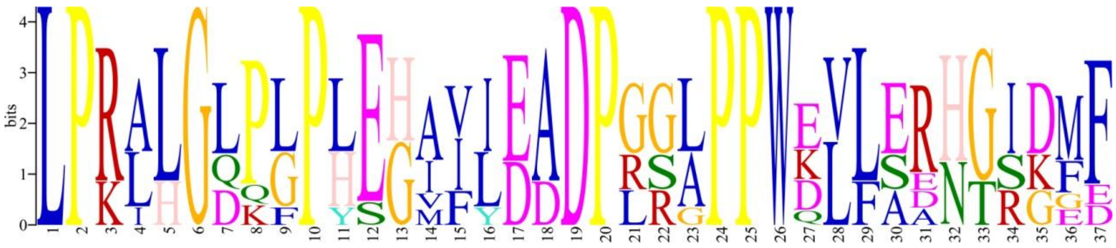

Motif 9

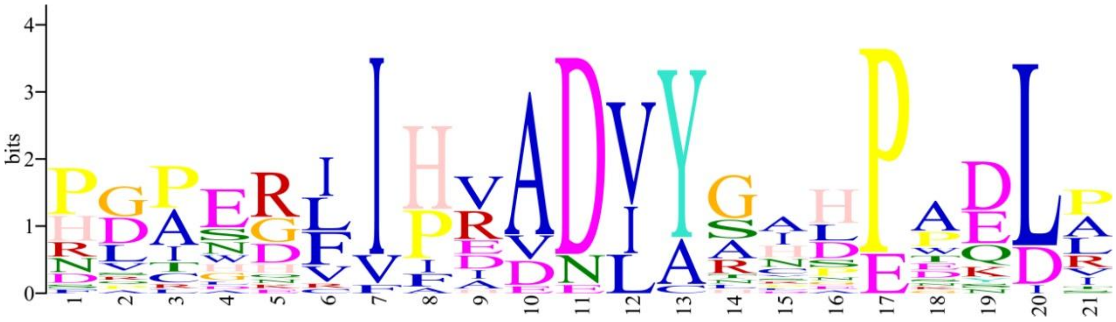

Motif 10

S2 Fig. Schematic diagram of NAC protein motifs in *Brachypodium*.
