# Supplementary figures and images for: Molecular Characterization and Expression Profiling of NAC Transcription Factors in Brachypodium distachyon L
Source: PLoS One. 2015 Oct 7;10(10):e0139794. doi: 10.1371/journal.pone.0139794 (PMC4596864; doi:10.1371/journal.pone.0139794)

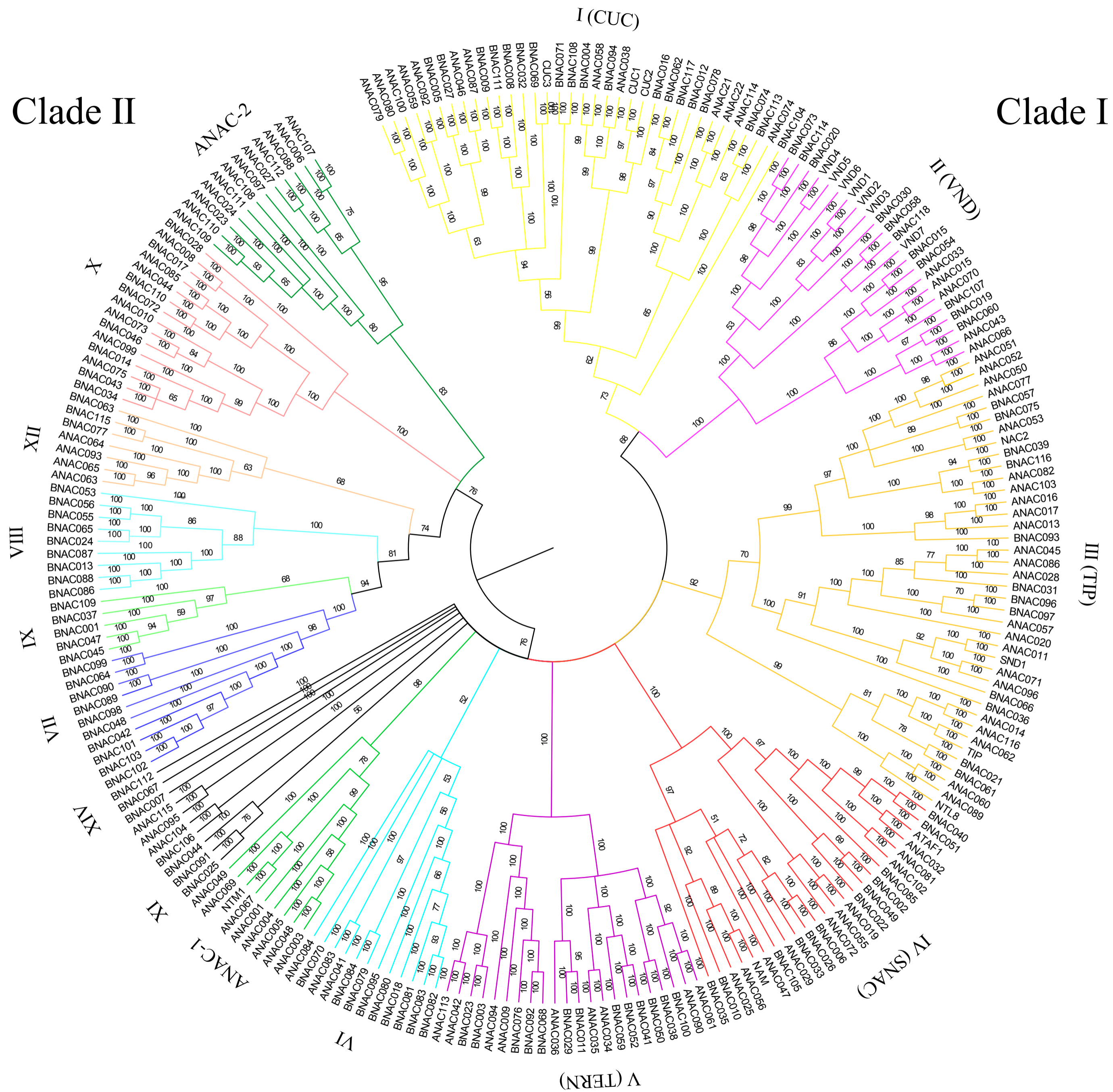

Supplement: S3 Fig — The amino acid sequences of 118 BNAC and 115 ANAC genes were aligned using MUSCLE program and the trees were generated based on Bayesian inference using Markov Chain Monte Carlo (MCMC) methods. (PDF) [file pone.0139794.s003.pdf]
