# Supplementary material for: Molecular Characterization and Expression Profiling of NAC Transcription Factors in Brachypodium distachyon L
Source: PLoS One. 2015 Oct 7;10(10):e0139794. doi: 10.1371/journal.pone.0139794 (PMC4596864; doi:10.1371/journal.pone.0139794)
Supplement: S4 Fig — The methods generating the diagram were based on those used in BNAC genes. A detailed motif introduction is shown in S5 Fig. (PDF) [file pone.0139794.s004.pdf]

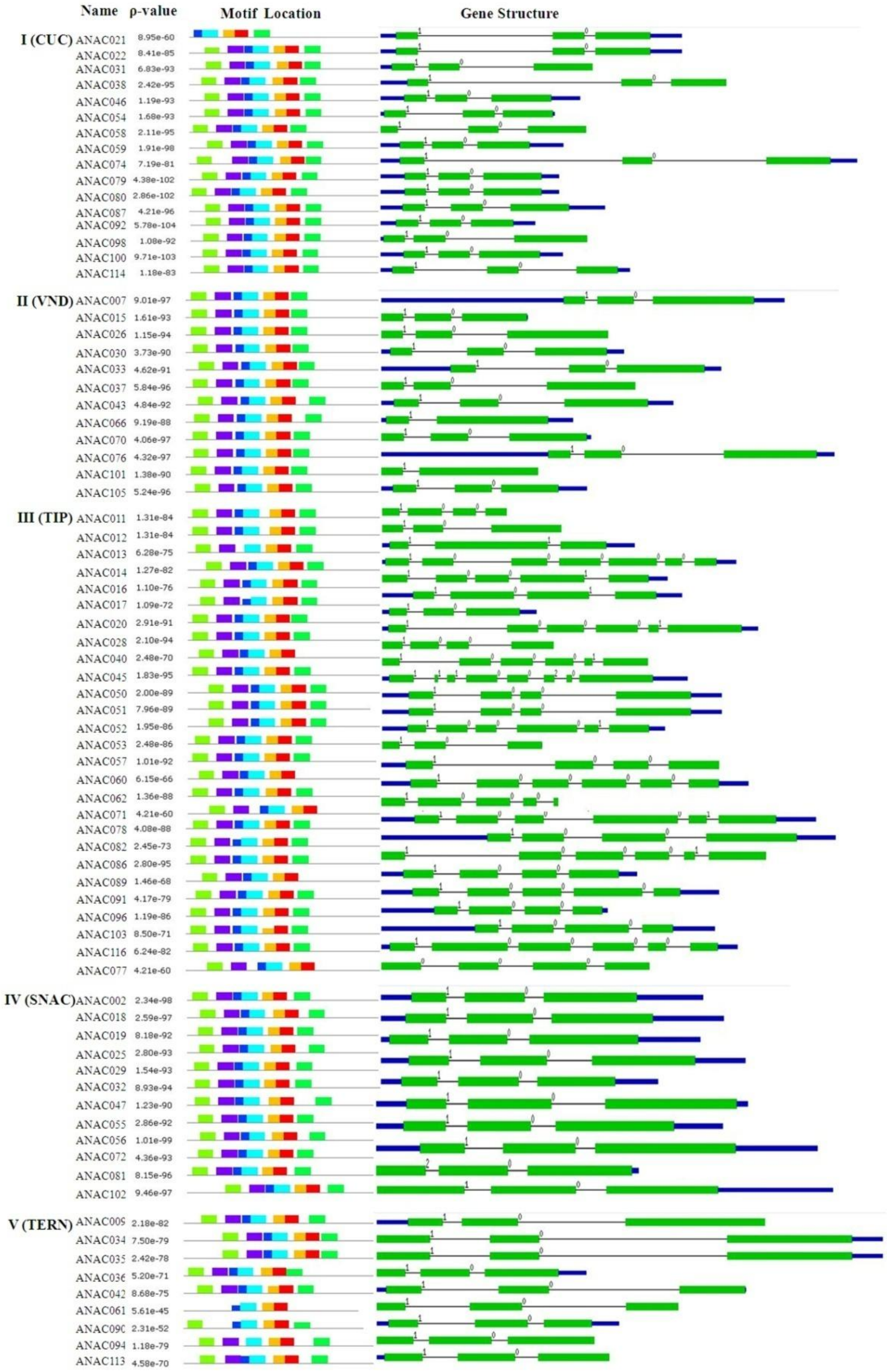
Name ρ-value
Motif Location
Gene Structure
I (CUC) ANAC021 8.95e-60
ANAC022 8.41e-85
ANAC031 6.83e-93
ANAC038 2.42e-95
ANAC046 1.19e-93
ANAC054 1.68e-93
ANAC058 2.11e-95
ANAC059 1.91e-98
ANAC074 7.19e-81
ANAC079 4.38e-102
ANAC080 2.86e-102
ANAC087 4.21e-96
ANAC092 5.78e-104
ANAC098 1.08e-92
ANAC100 9.71e-103
ANAC114 1.18e-83
II (VND) ANAC007 9.01e-97
ANAC015 1.61e-93
ANAC026 1.15e-94
ANAC030 3.73e-90
ANAC033 4.62e-91
ANAC037 5.84e-96
ANAC043 4.84e-92
ANAC066 9.19e-88
ANAC070 4.06e-97
ANAC076 4.32e-97
ANAC101 1.38e-90
ANAC105 5.24e-96
III (TIP) ANAC011 1.31e-84
ANAC012 1.31e-84
ANAC013 6.28e-75
ANAC014 1.27e-82
ANAC016 1.10e-76
ANAC017 1.09e-72
ANAC020 2.91e-91
ANAC028 2.10e-94
ANAC040 2.48e-70
ANAC045 1.83e-95
ANAC050 2.00e-89
ANAC051 7.96e-89
ANAC052 1.95e-86
ANAC053 2.48e-86
ANAC057 1.01e-92
ANAC060 6.15e-66
ANAC062 1.36e-88
ANAC071 4.21e-60
ANAC078 4.08e-88
ANAC082 2.45e-73
ANAC086 2.80e-95
ANAC089 1.46e-68
ANAC091 4.17e-79
ANAC096 1.19e-86
ANAC103 8.50e-71
ANAC116 6.24e-82
ANAC077 4.21e-60
IV (SNAC) ANAC002 2.34e-98
ANAC018 2.59e-97
ANAC019 8.18e-92
ANAC025 2.80e-93
ANAC029 1.54e-93
ANAC032 8.93e-94
ANAC047 1.23e-90
ANAC055 2.86e-92
ANAC056 1.01e-99
ANAC072 4.36e-93
ANAC081 8.15e-96
ANAC102 9.46e-97
V (TERN) ANAC009 2.18e-82
ANAC034 7.50e-79
ANAC035 2.42e-78
ANAC036 5.20e-71
ANAC042 8.68e-75
ANAC061 5.61e-45
ANAC090 2.31e-52
ANAC094 1.18e-79
ANAC113 4.58e-70

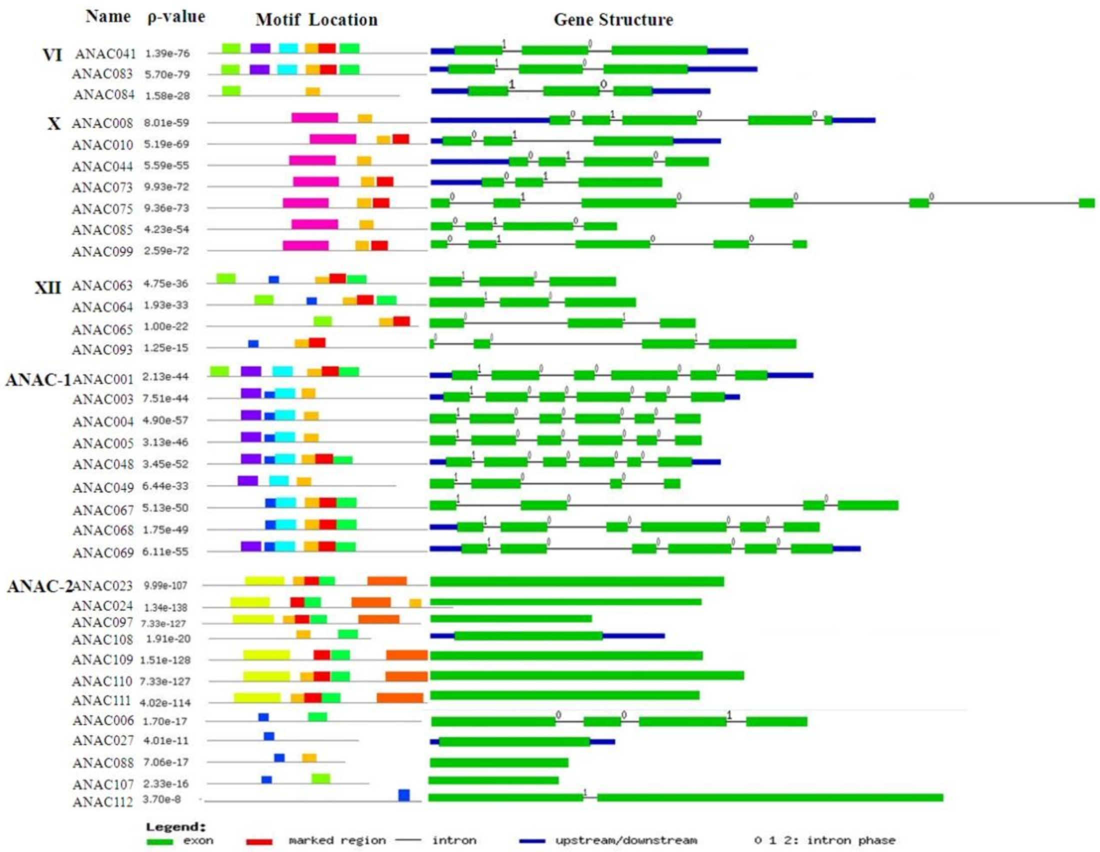

S4 Fig. Conserved motifs and exon-intron organizations of *Arabidopsis* NAC genes. The methods generating the diagram were based on those used in BNAC genes. A detailed motif introduction is shown in S5 Fig.
