# Supplementary material for: Molecular Characterization and Expression Profiling of NAC Transcription Factors in Brachypodium distachyon L
Source: PLoS One. 2015 Oct 7;10(10):e0139794. doi: 10.1371/journal.pone.0139794 (PMC4596864; doi:10.1371/journal.pone.0139794)
Supplement: S6 Fig — The green boxes represent exons, the black solid lines connecting two neighboring exons represent introns and the blue boxes represent 5’-UTR and 3’-UTR. The grid scales show the gene sizes (kb). (PDF) [file pone.0139794.s006.pdf]

Clade 1

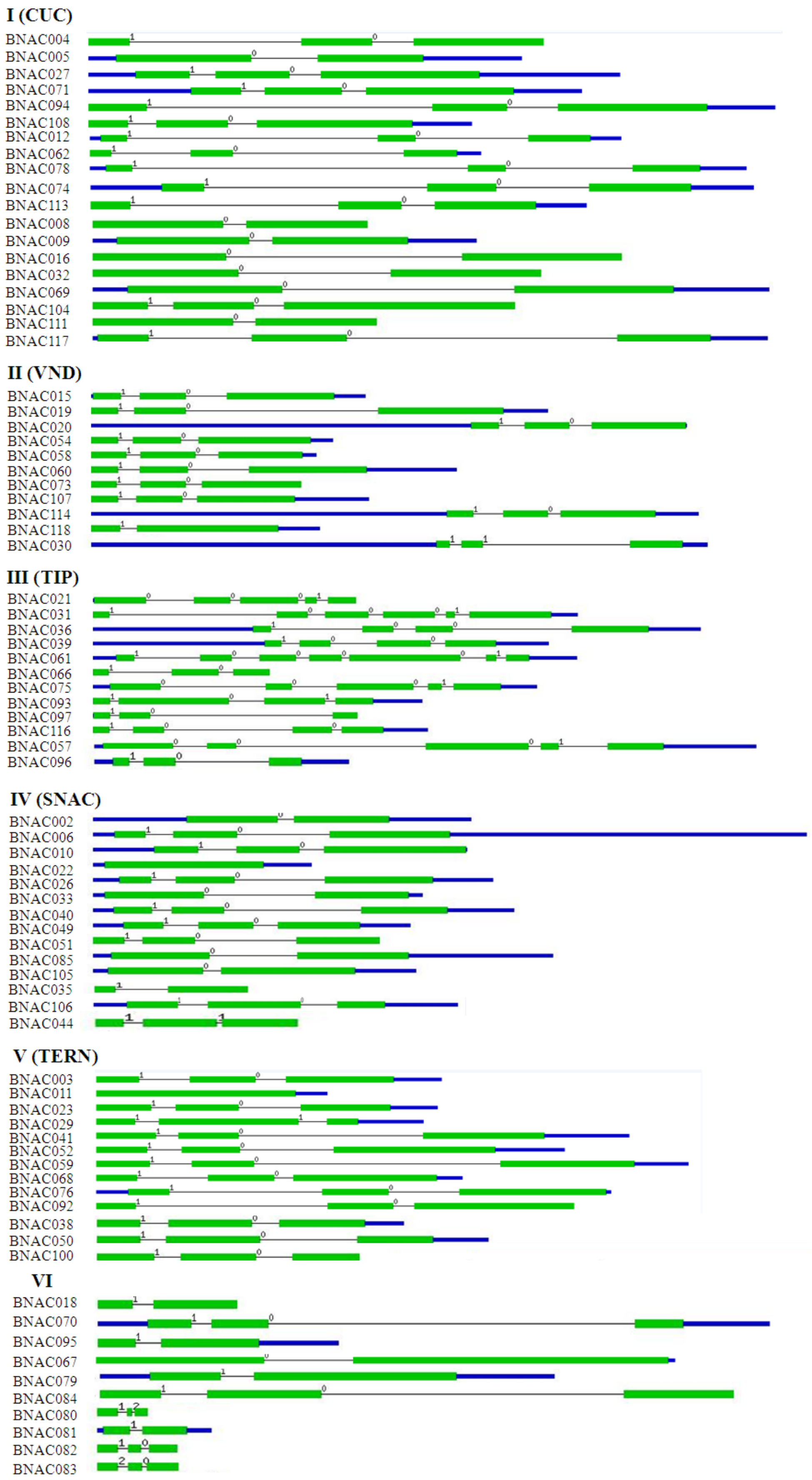
I (CUC)
BNAC004
BNAC005
BNAC027
BNAC071
BNAC094
BNAC108
BNAC012
BNAC062
BNAC078
BNAC074
BNAC113
BNAC008
BNAC009
BNAC016
BNAC032
BNAC069
BNAC104
BNAC111
BNAC117
II (VND)
BNAC015
BNAC019
BNAC020
BNAC054
BNAC058
BNAC060
BNAC073
BNAC107
BNAC114
BNAC118
BNAC030
III (TIP)
BNAC021
BNAC031
BNAC036
BNAC039
BNAC061
BNAC066
BNAC075
BNAC093
BNAC097
BNAC116
BNAC057
BNAC096
IV (SNAC)
BNAC002
BNAC006
BNAC010
BNAC022
BNAC026
BNAC033
BNAC040
BNAC049
BNAC051
BNAC085
BNAC105
BNAC035
BNAC106
BNAC044
V (TERN)
BNAC003
BNAC011
BNAC023
BNAC029
BNAC041
BNAC052
BNAC059
BNAC068
BNAC076
BNAC092
BNAC038
BNAC050
BNAC100
VI
BNAC018
BNAC070
BNAC095
BNAC067
BNAC079
BNAC084
BNAC080
BNAC081
BNAC082
BNAC083

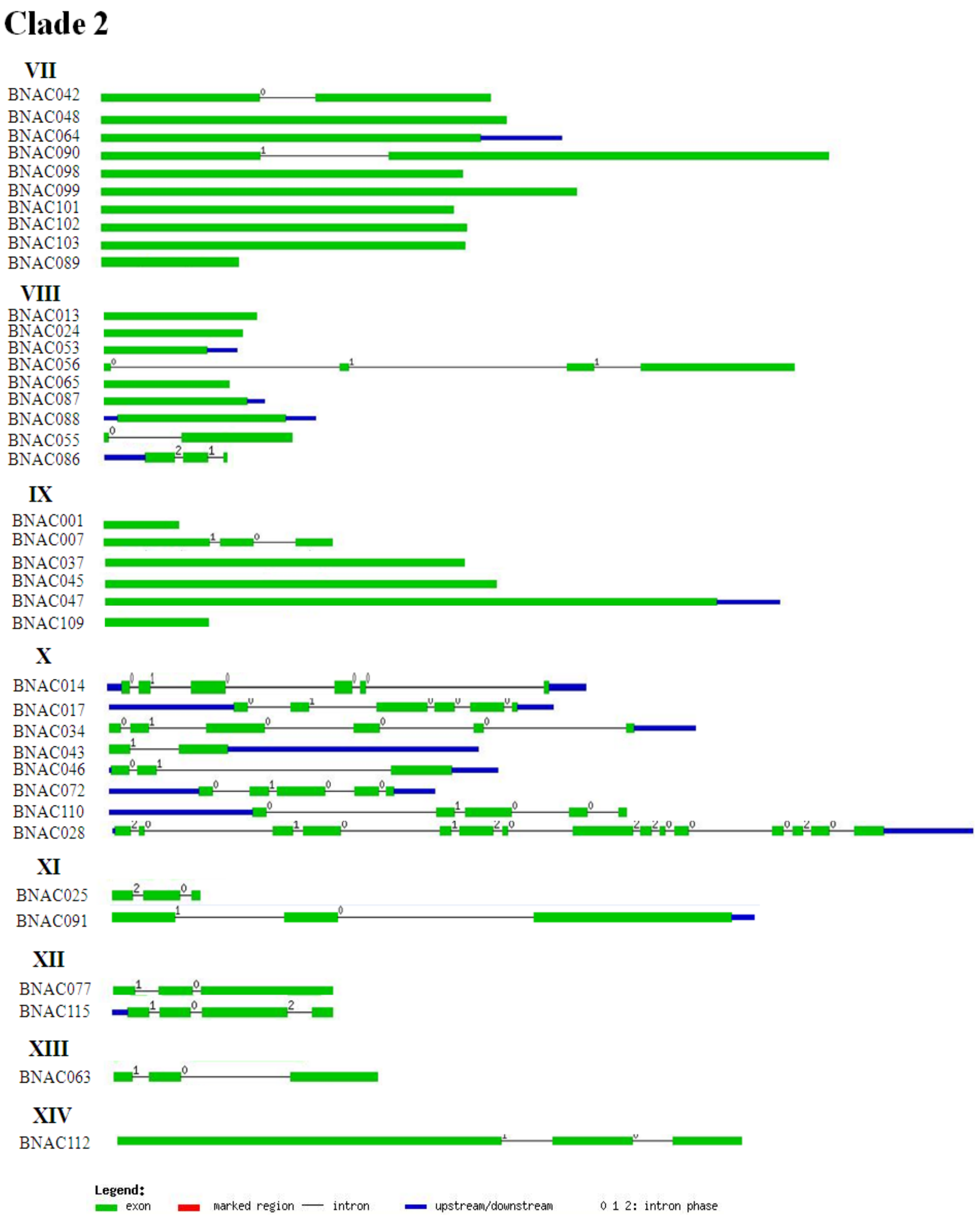

S6 Fig. Exon-intron structures of *B. distachyon* NAC genes. The green boxes represent exons, the black solid lines connecting two neighboring exons represent introns and the blue boxes represent 5'-UTR and 3'-UTR. The grid scales show the gene sizes (kb).
