# Supplementary material for: Molecular Characterization and Expression Profiling of NAC Transcription Factors in Brachypodium distachyon L
Source: PLoS One. 2015 Oct 7;10(10):e0139794. doi: 10.1371/journal.pone.0139794 (PMC4596864; doi:10.1371/journal.pone.0139794)
Supplement: S1 File — Of 23 BNAC genes under different abiotic stresses (Figure A). Of 6 representative BNAC genes during drought and salt stresses (Figure B). Of 6 representative BNAC genes during cold, gibberellin and H2O2 stresses (Figure C). The red standard curve represents reference genes and other blue curve represents BNAC genes. (PDF) [file pone.0139794.s007.pdf]

A

BNAC002

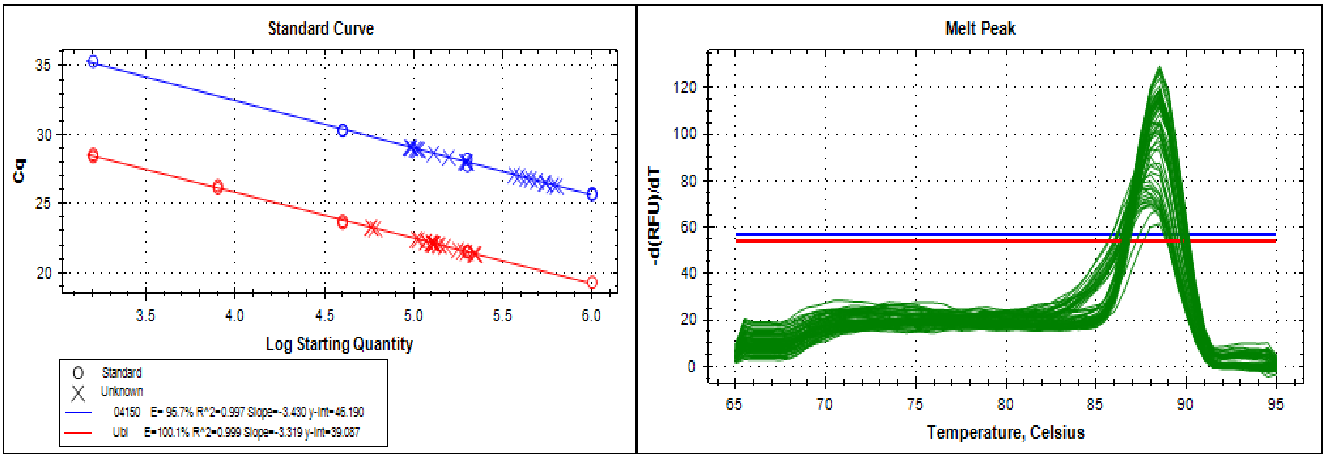

BNAC005

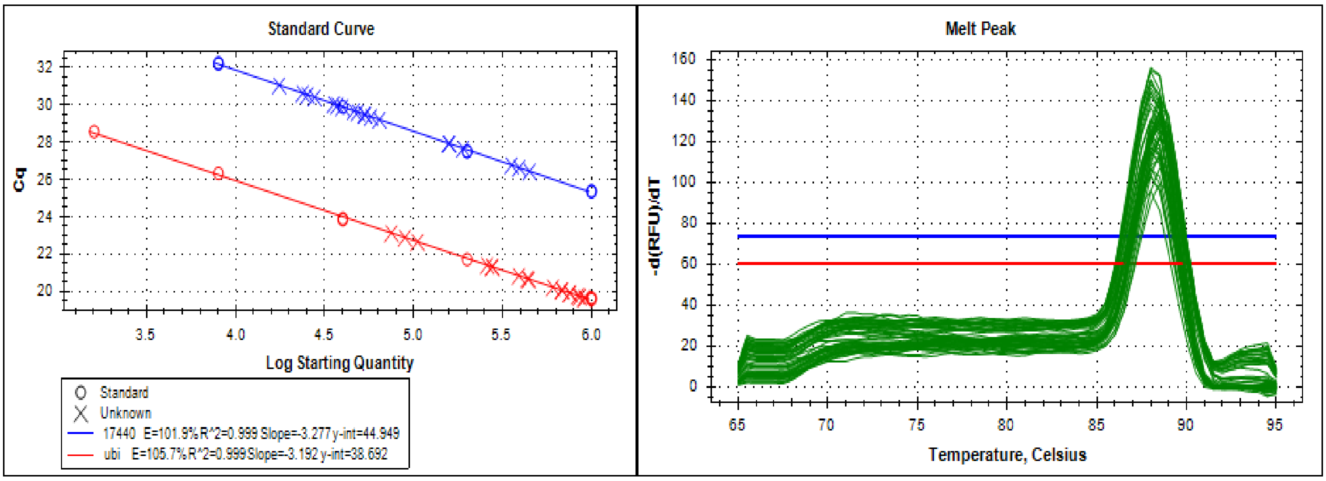

BNAC006

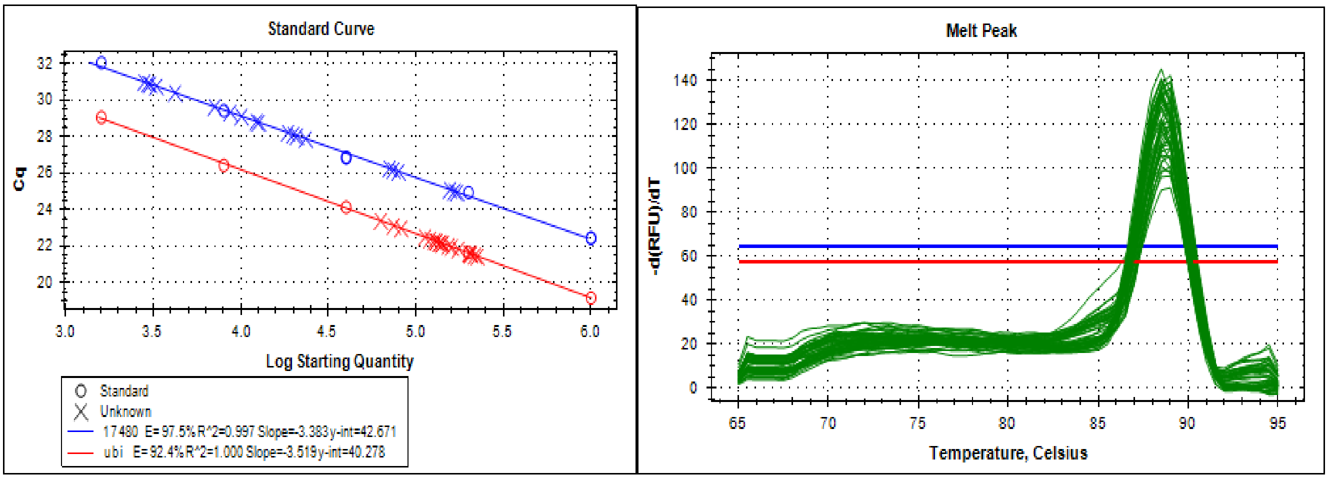

BNAC010

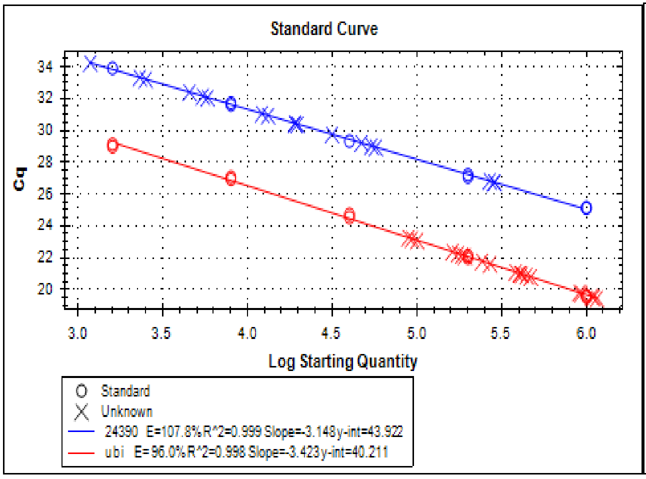

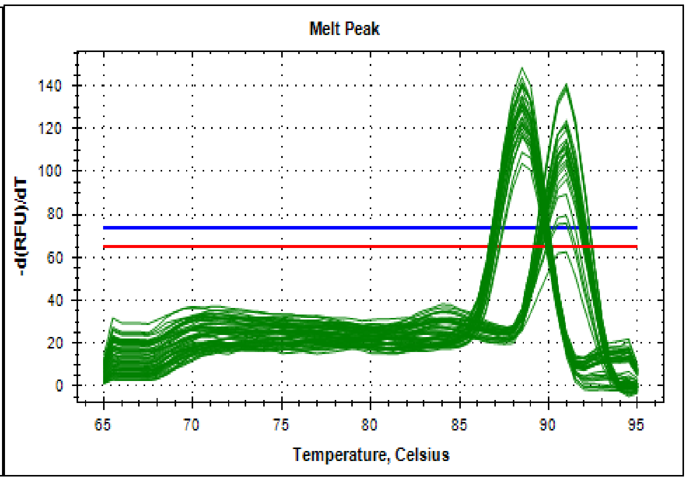

BNAC017

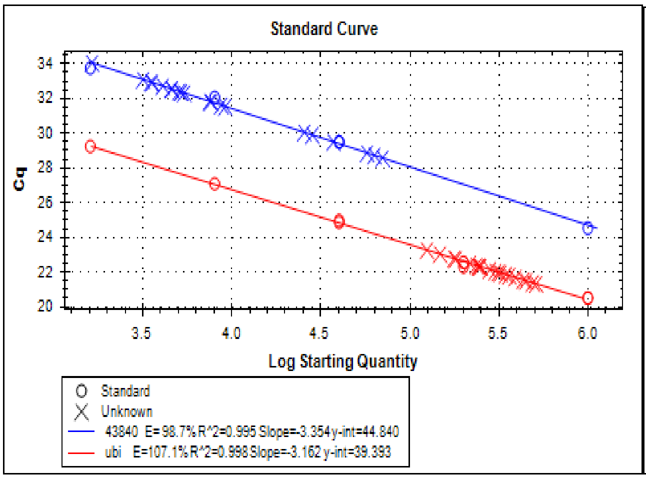

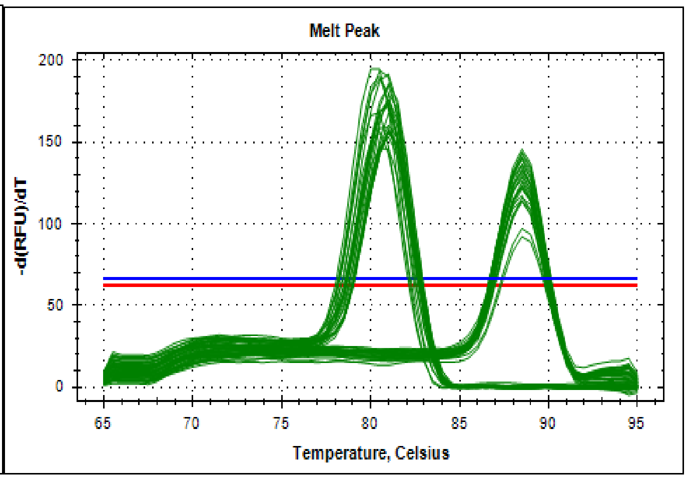

BNAC019

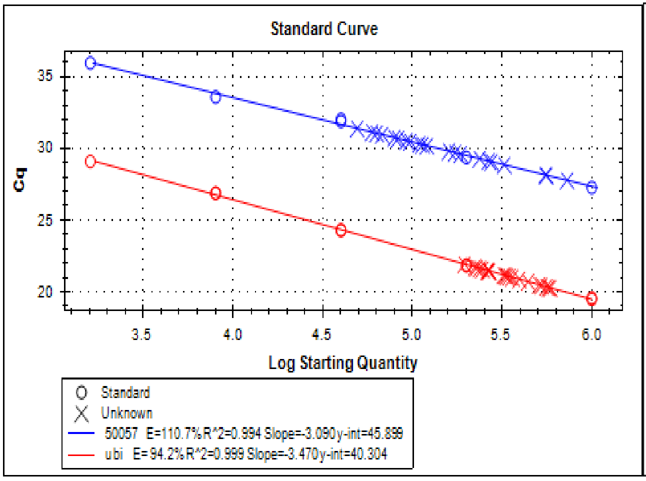

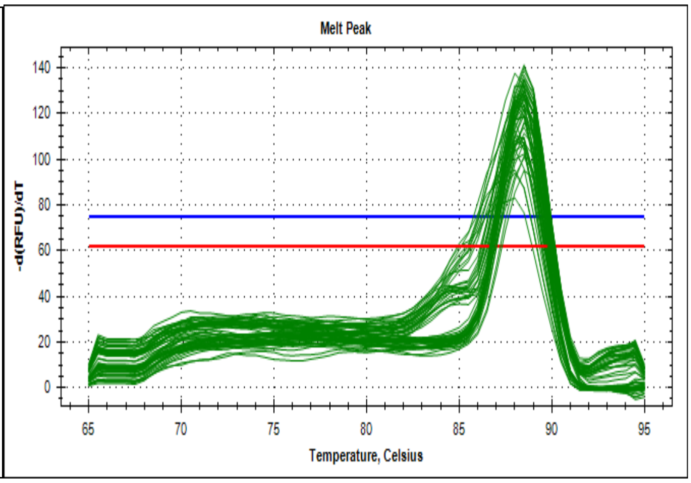

BNAC022

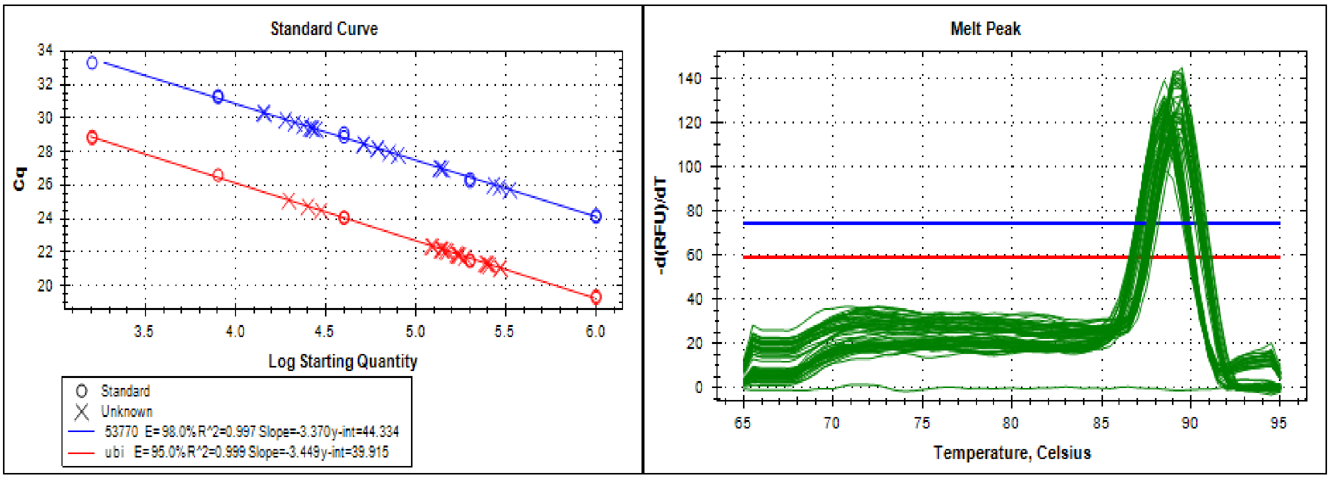

BNAC024

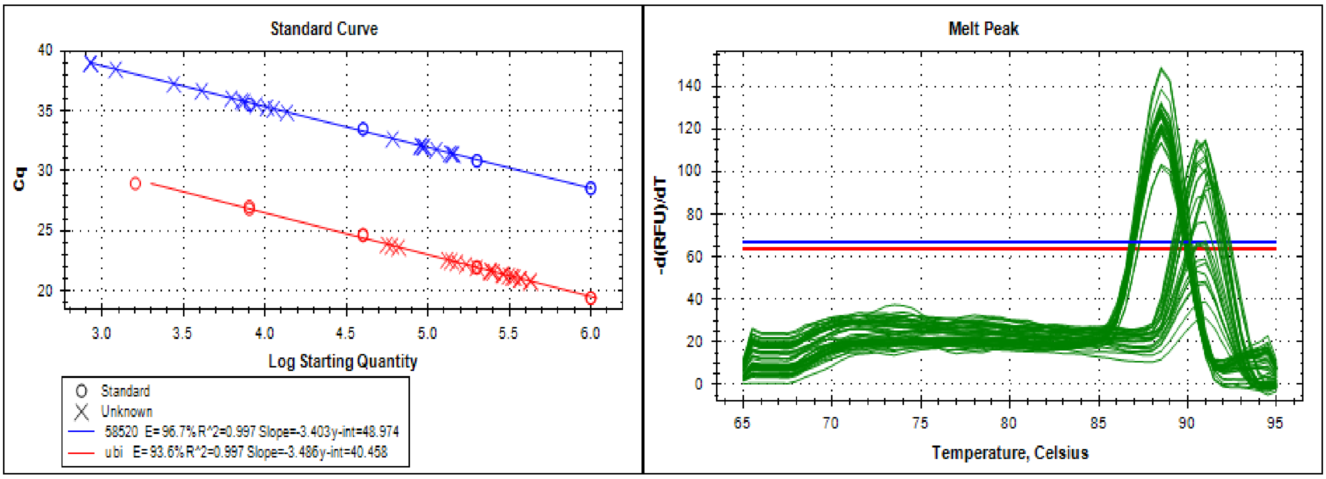

BNAC026

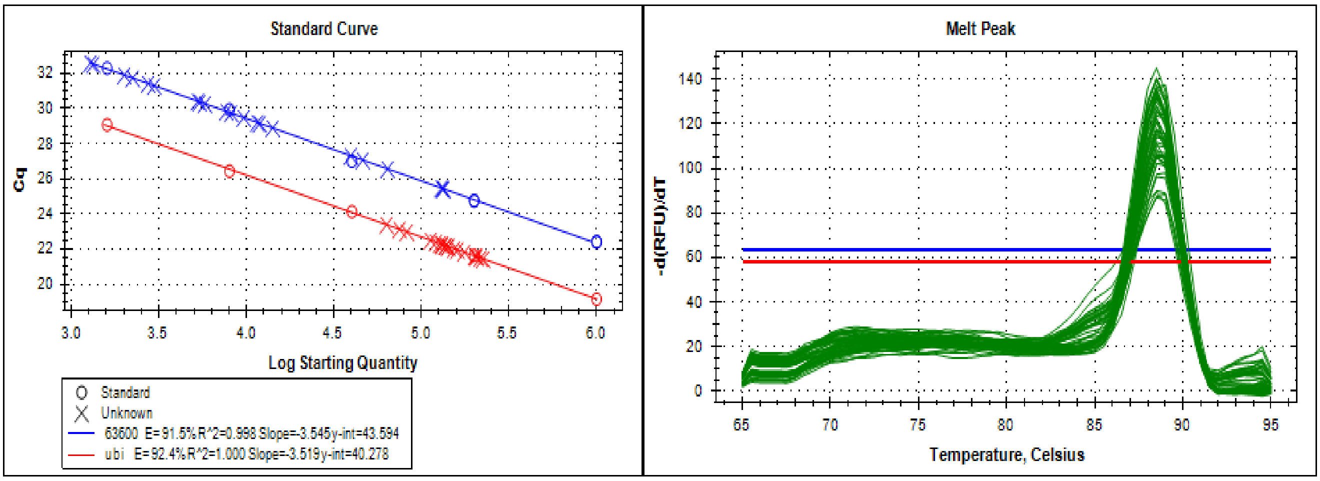

BNAC027

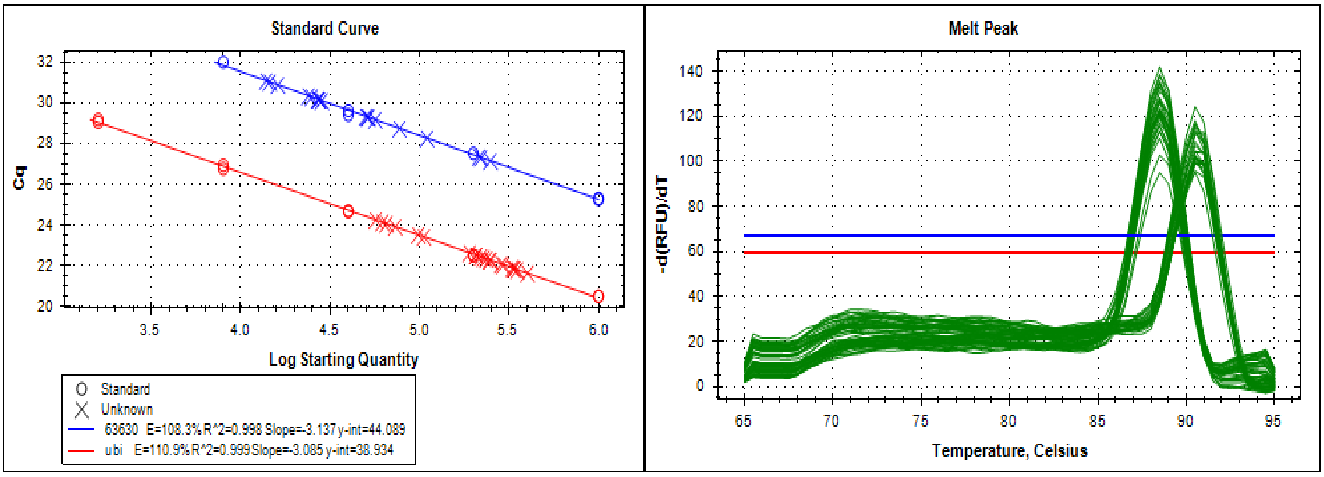

BNAC031

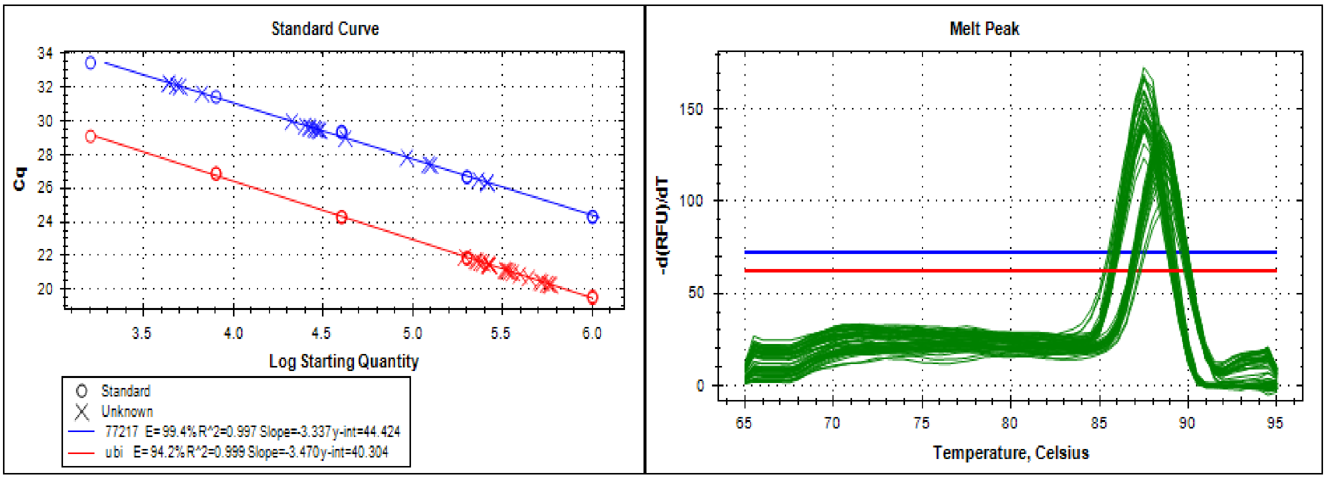

BNAC039

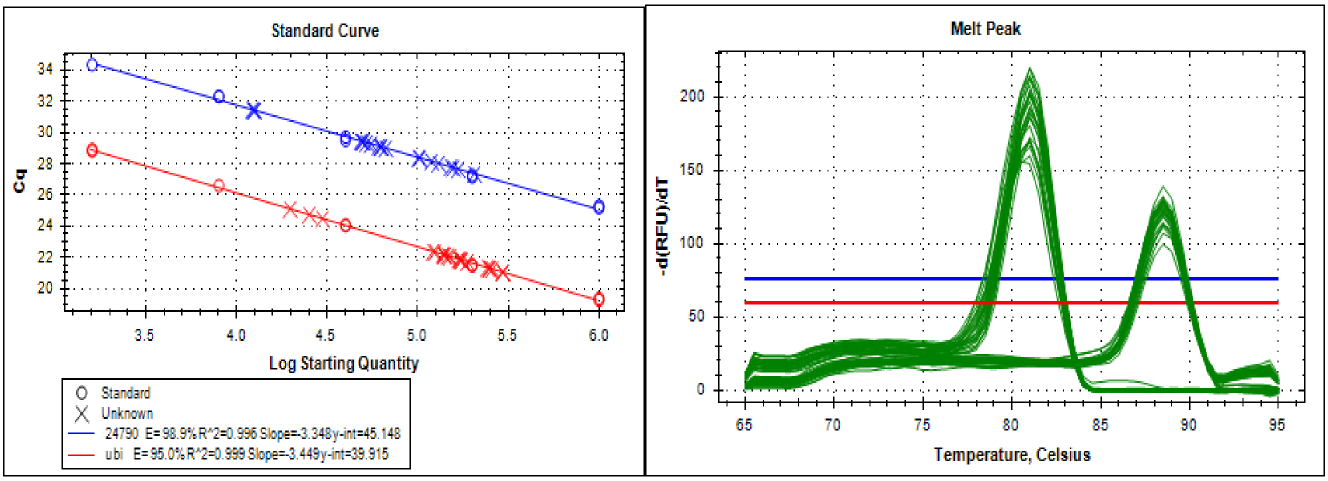

BNAC049

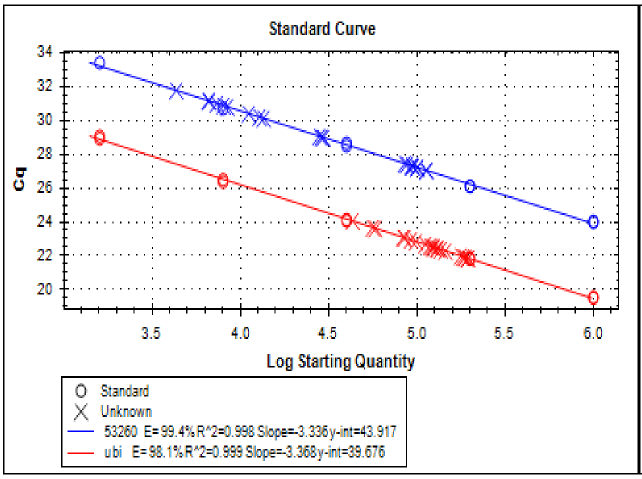

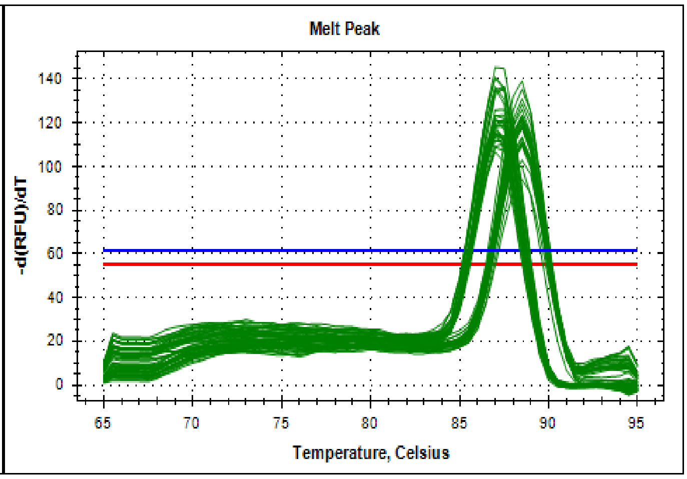

BNAC067

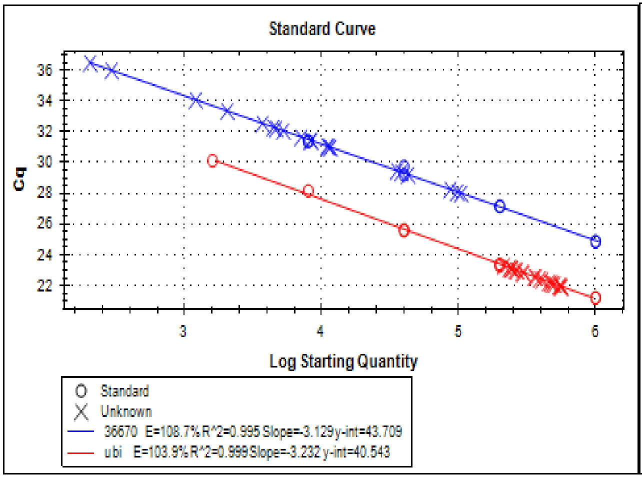

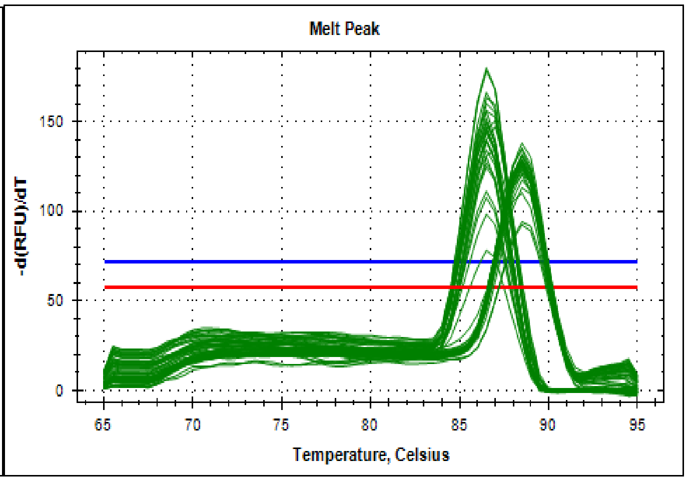

BNAC070

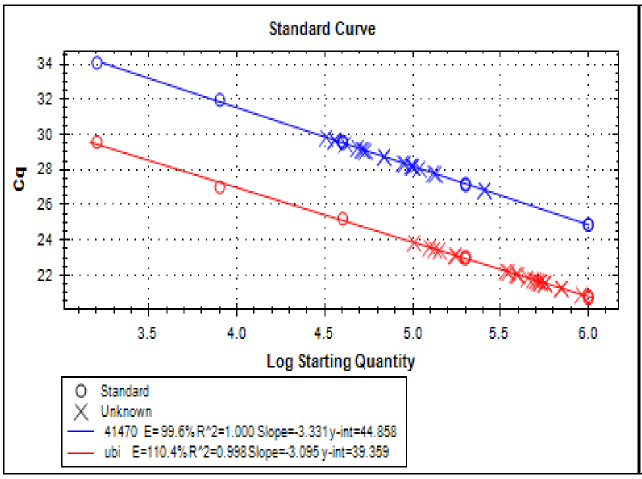

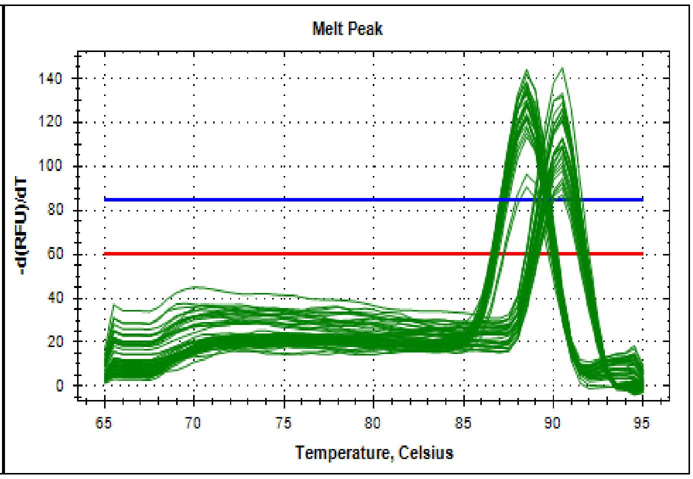

BNAC073

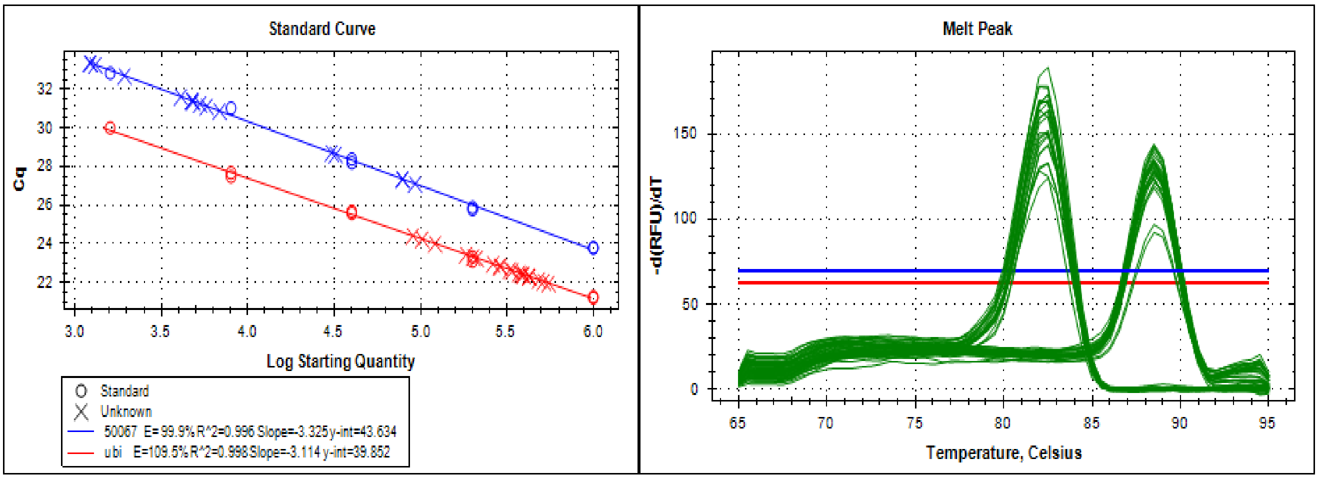

BNAC076

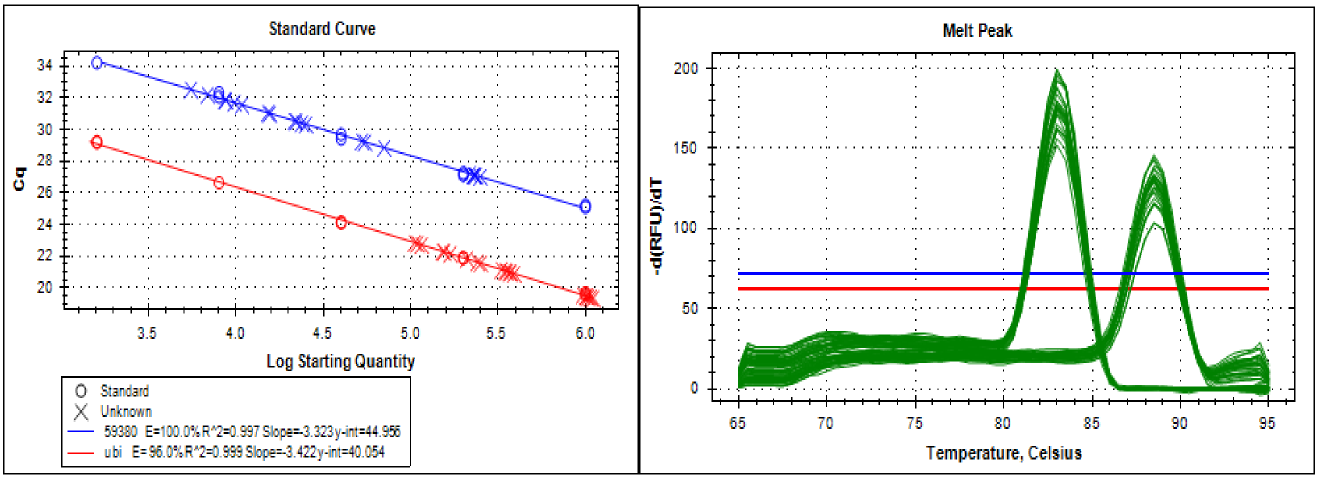

BNAC079

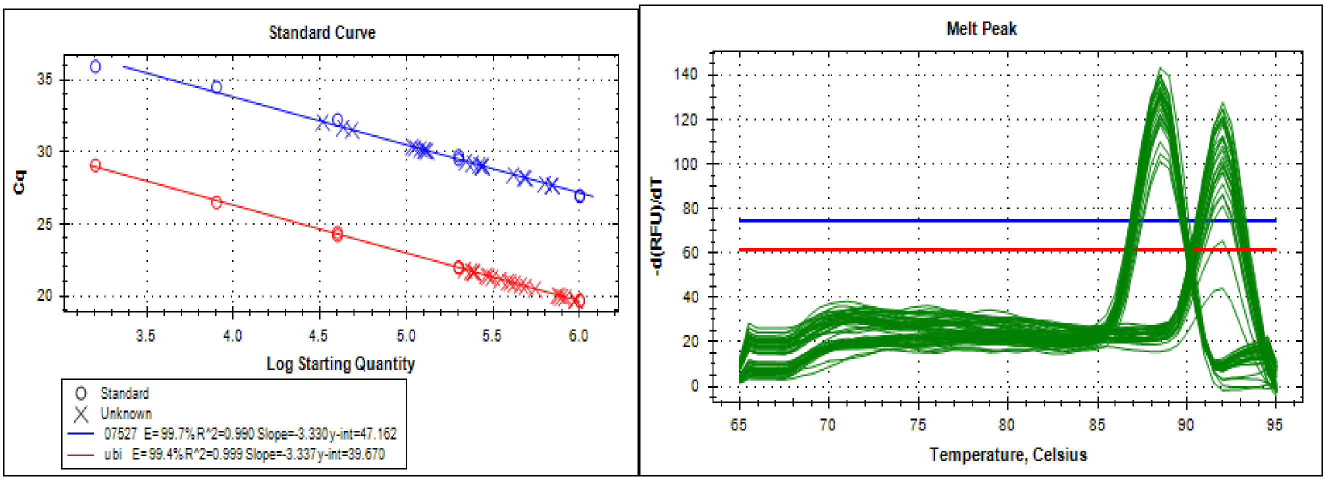

BNAC092

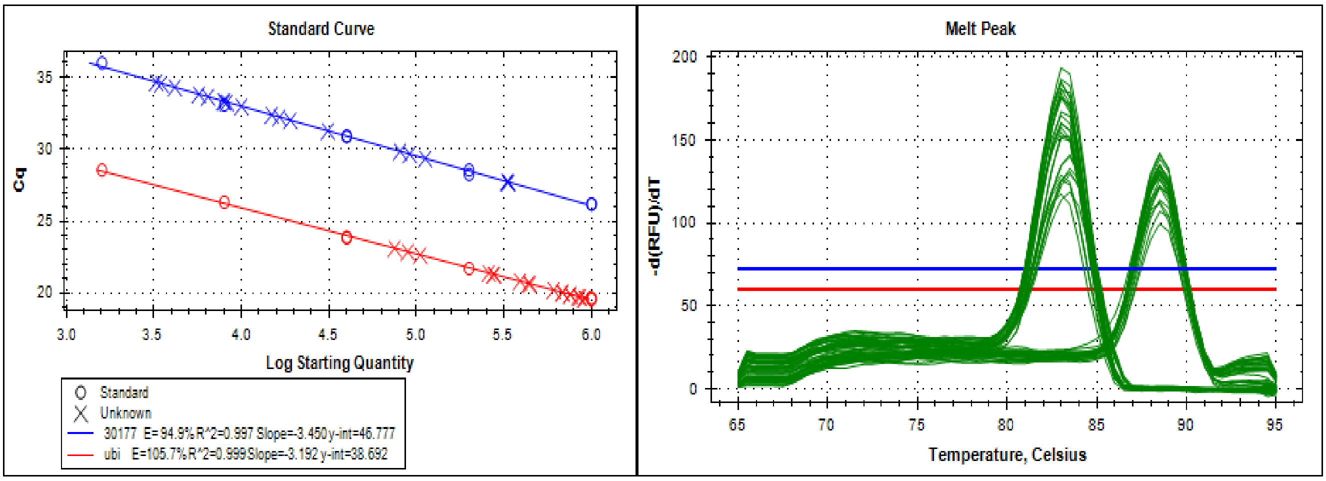

BNAC098

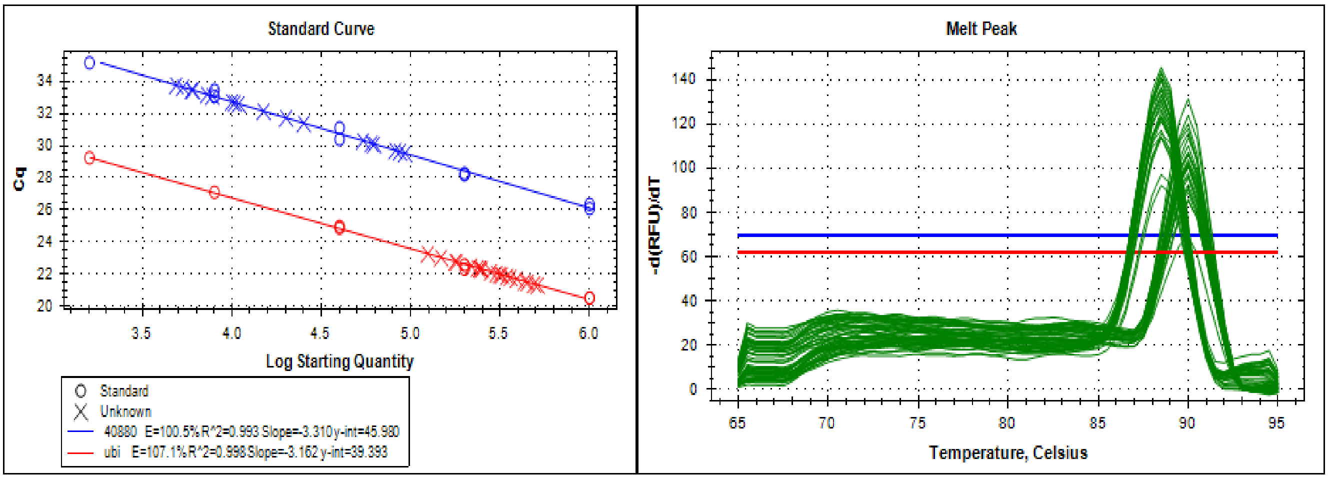

BNAC105

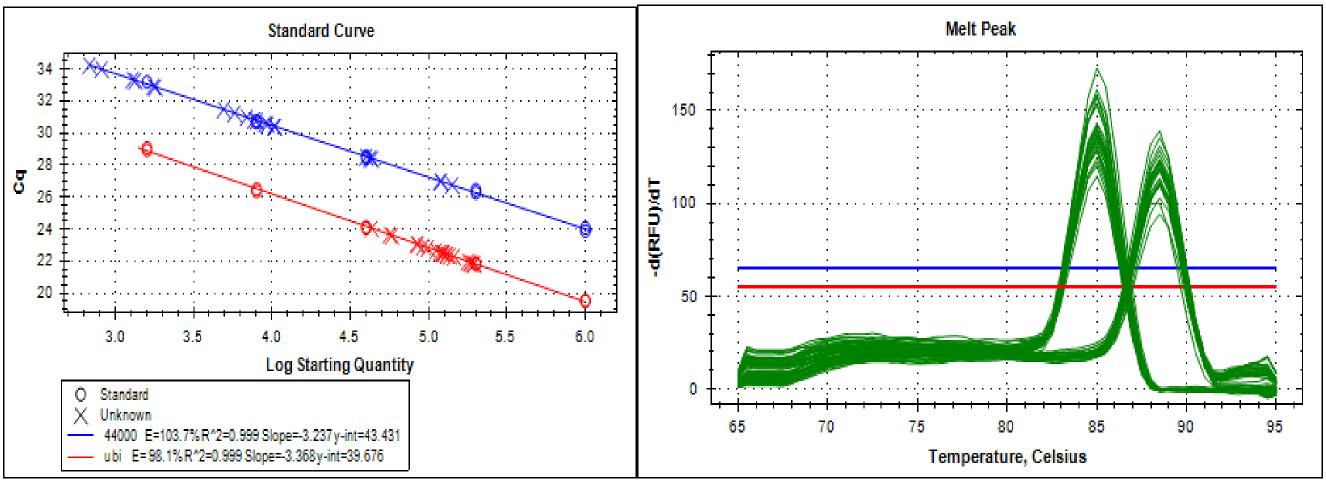

BNAC106

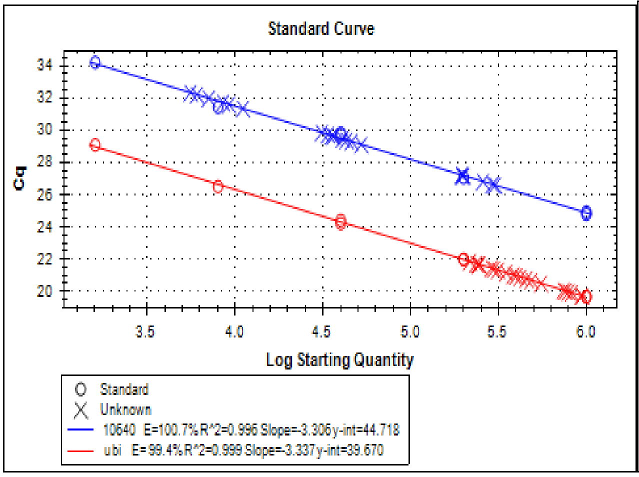

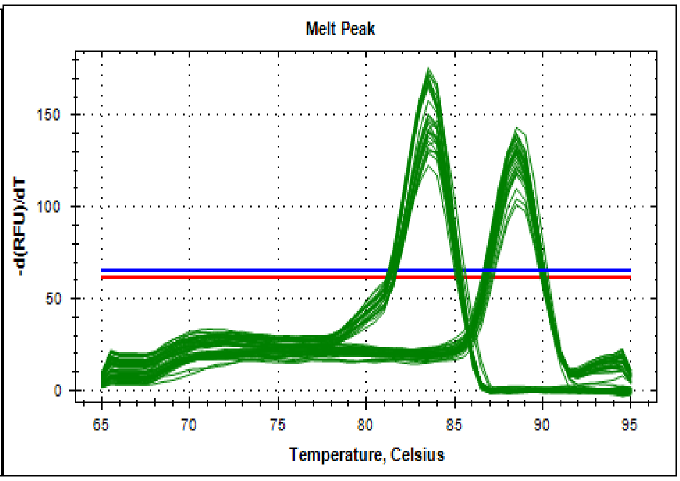

BNAC113

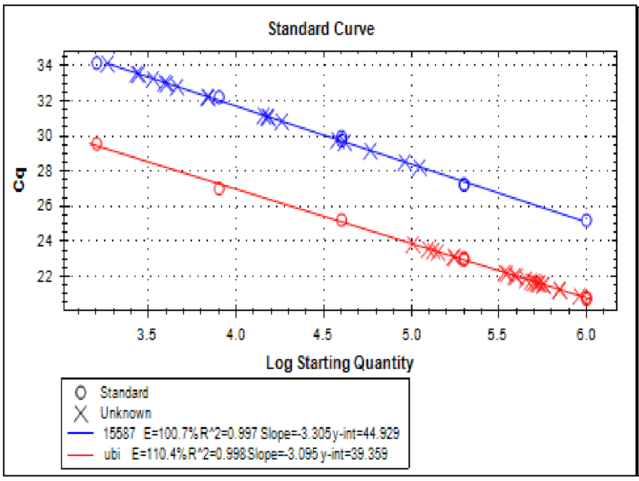

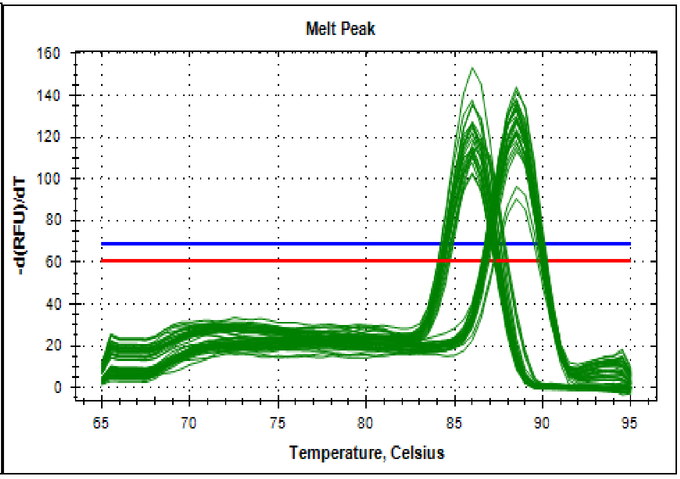

B

BNAC010

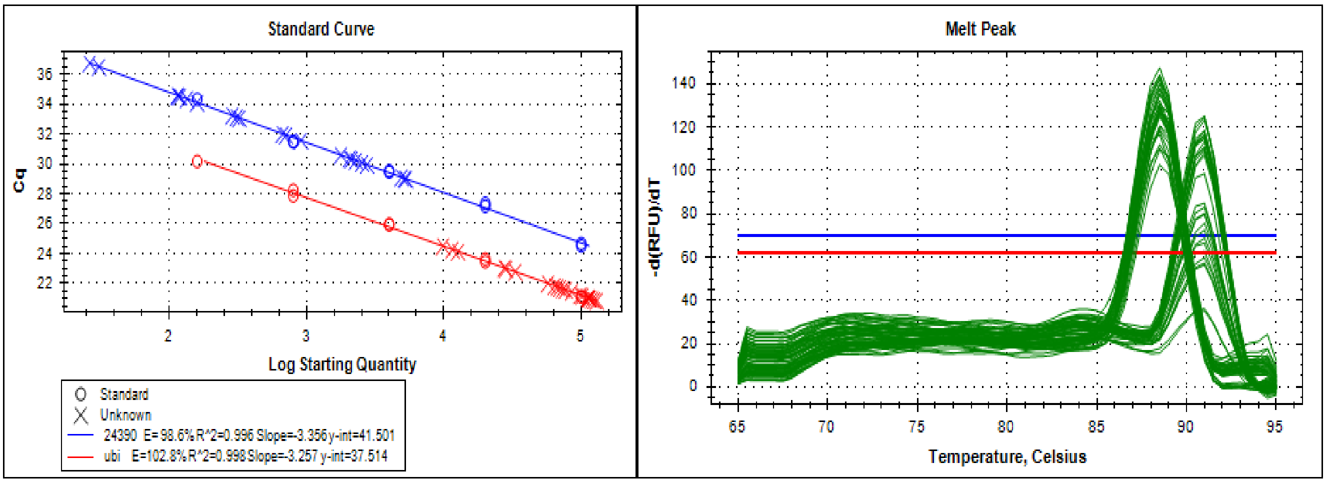

BNAC031

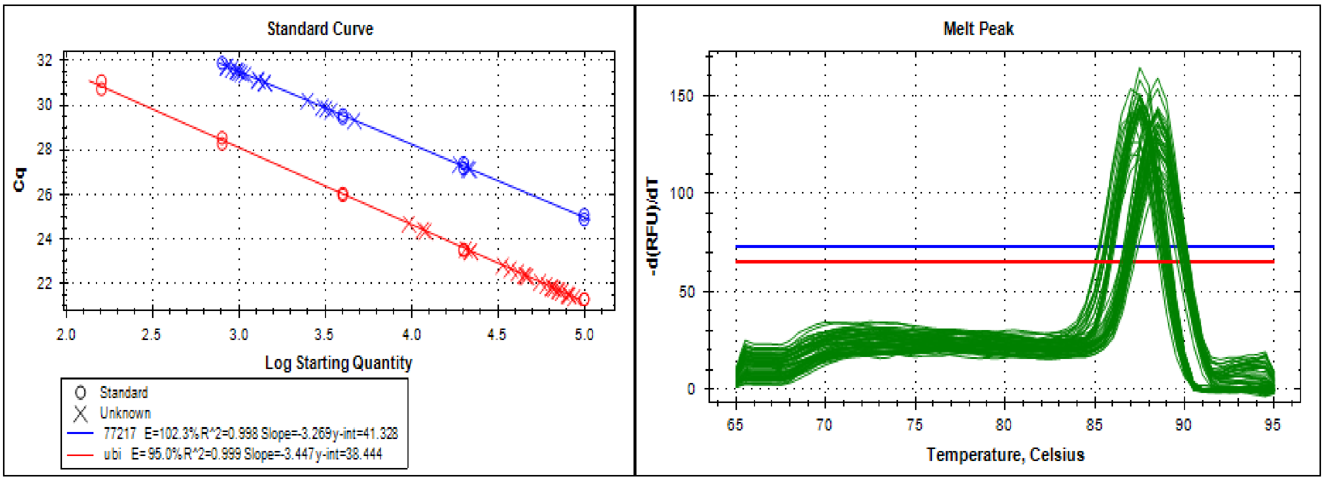

BNAC039

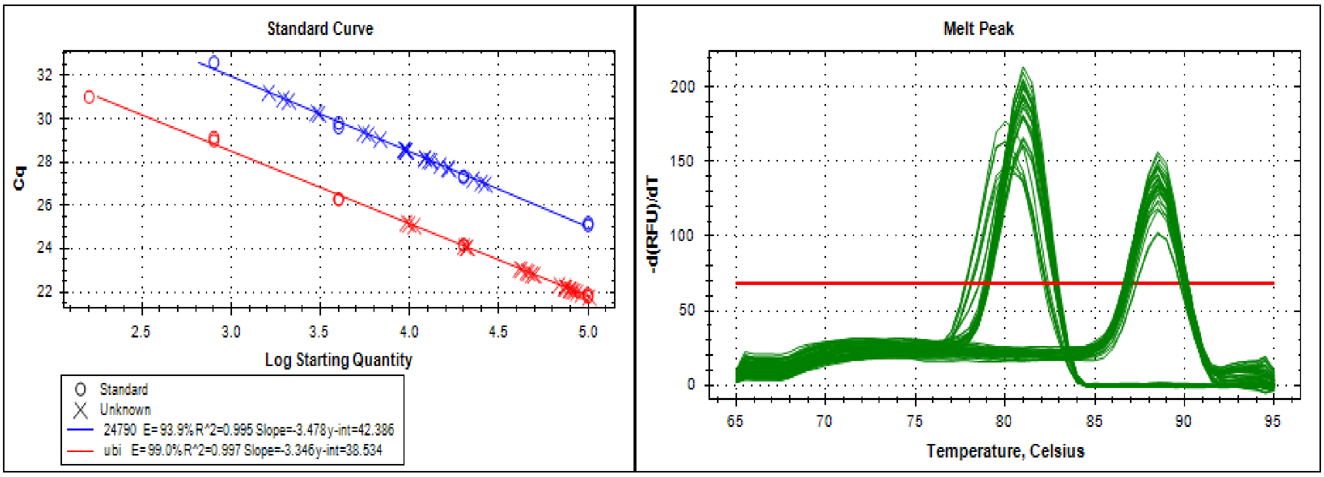

BNAC070

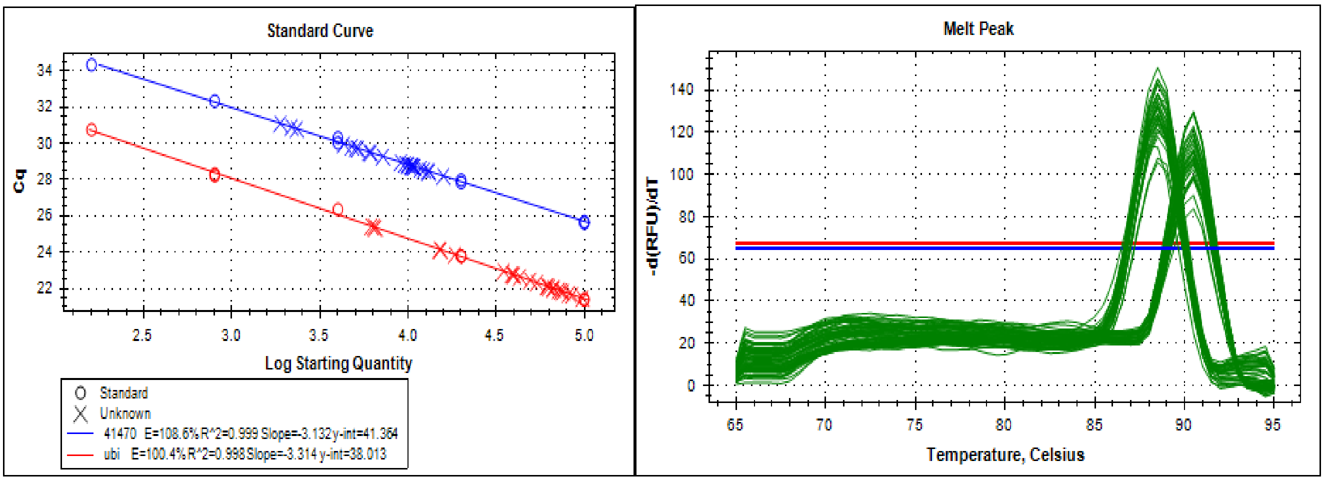

BNAC076

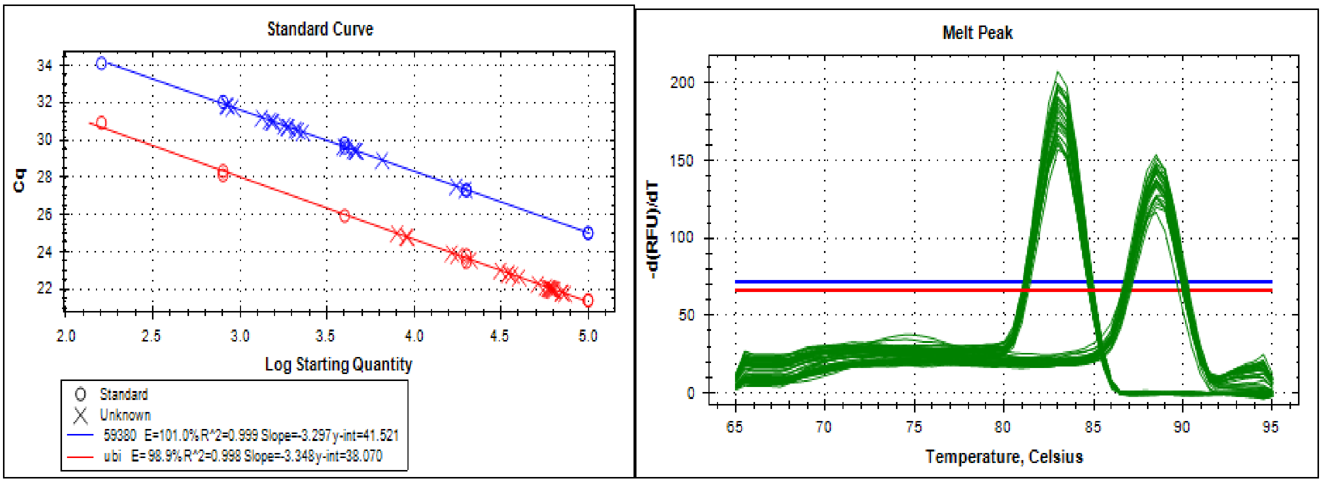

BNAC079

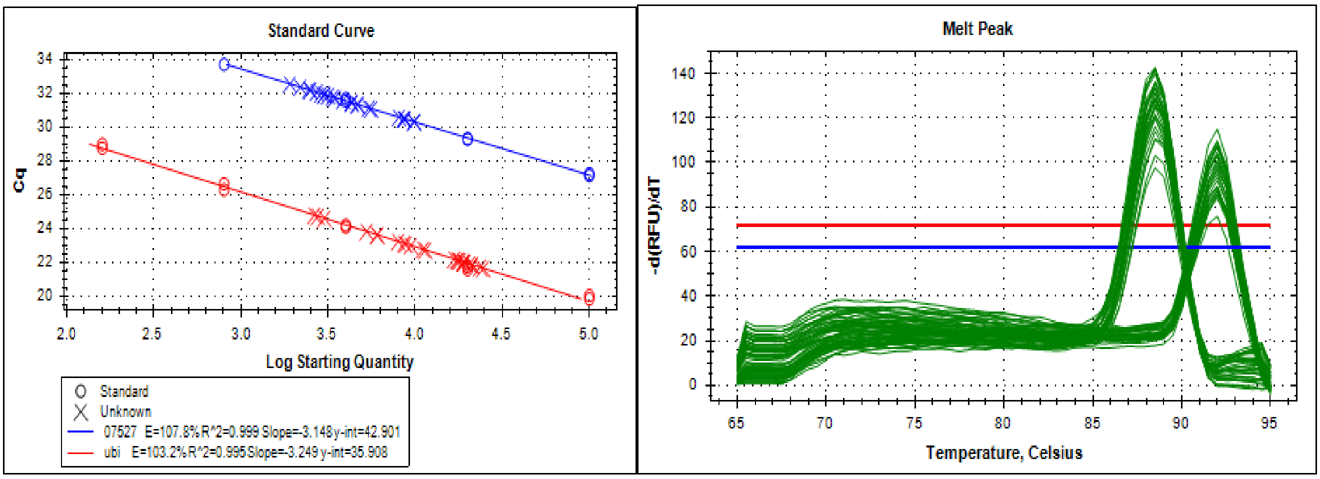

C

BNAC010

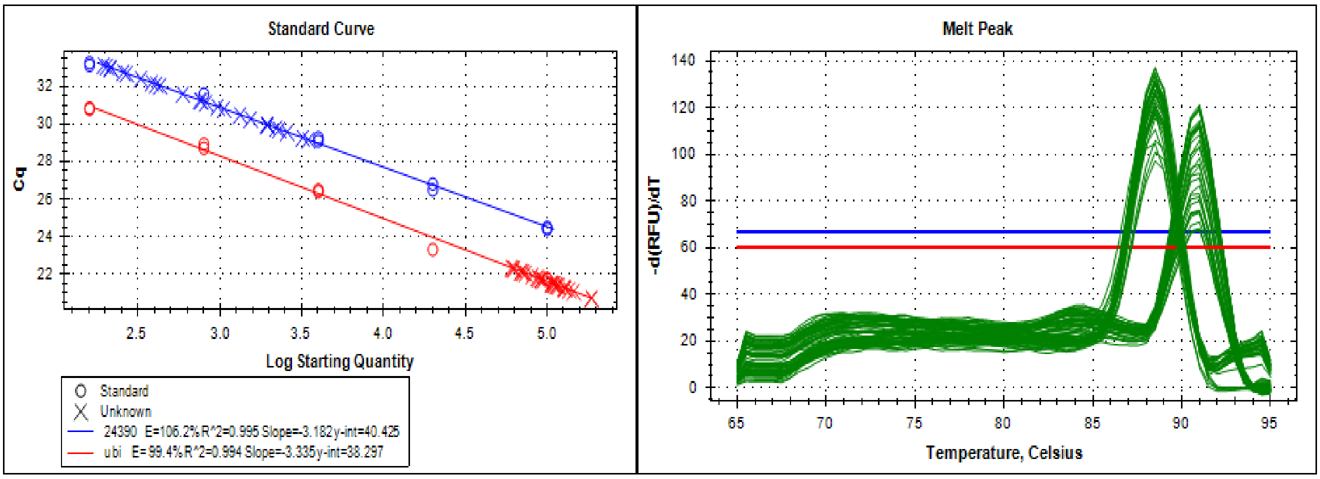

BNAC031

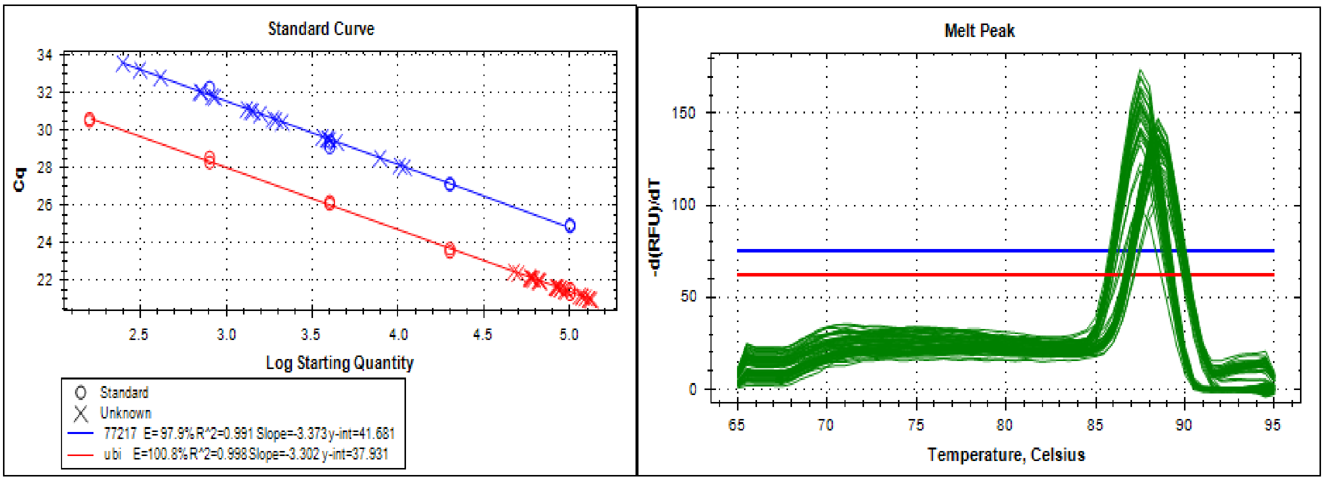

BNAC039

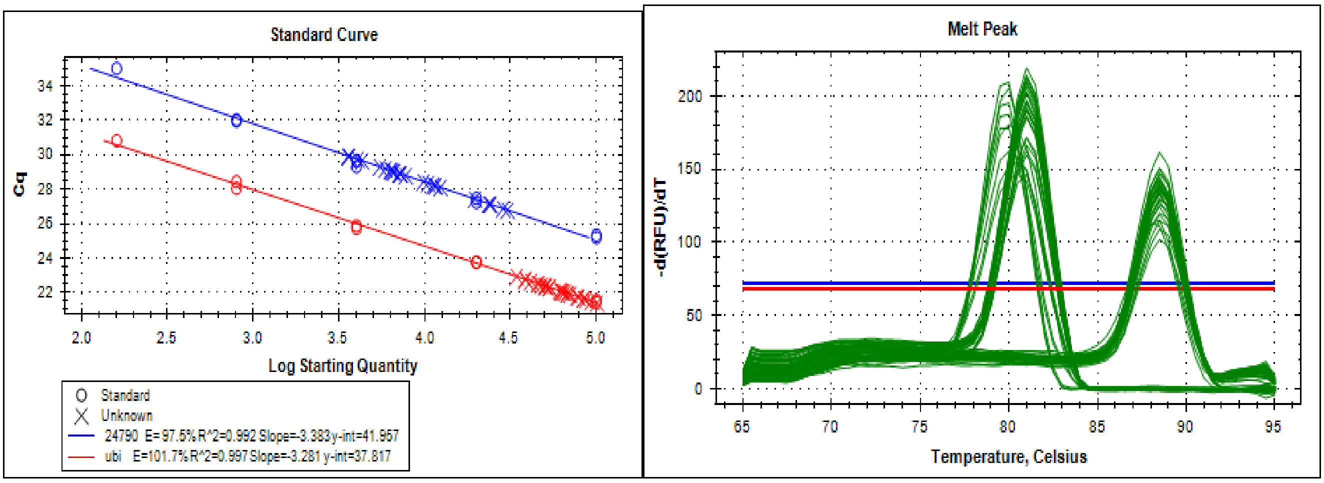

BNAC070

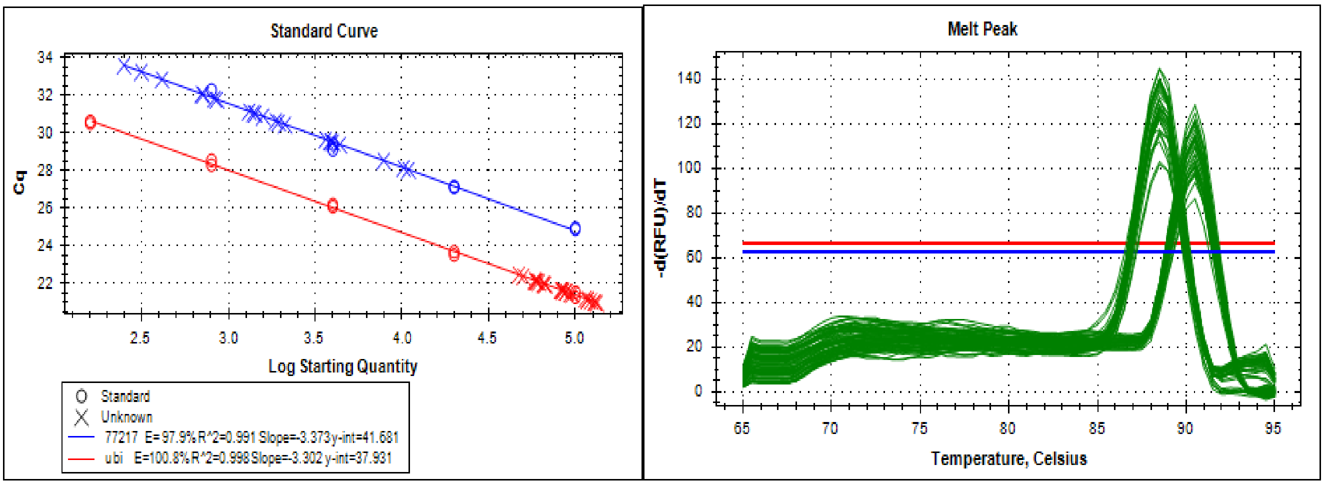

BNAC076

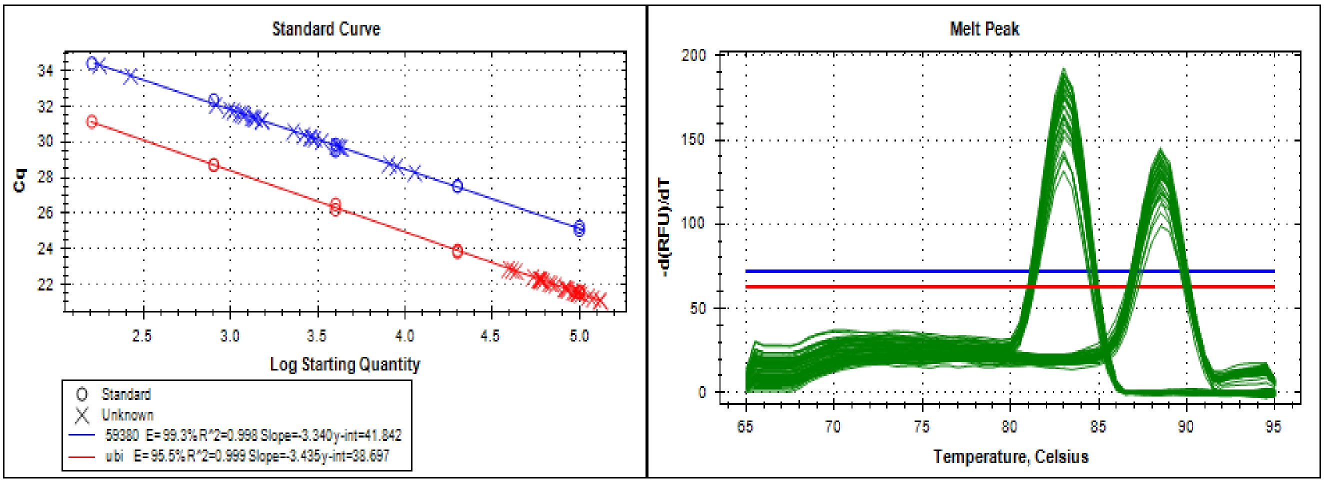

BNAC079

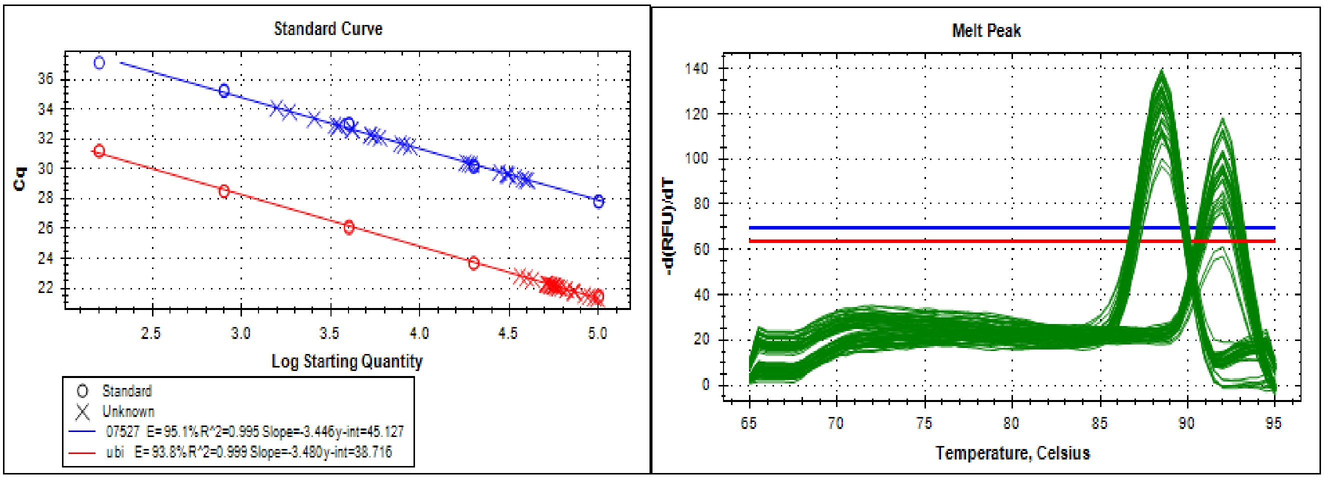

S1 File. Double standard curve and dissolution curve. Of 23 *BNAC* genes under different abiotic stresses **(Figure A)**. of 6 representative *BNAC* genes during drought and salt stresses **(Figure B)**. Of 6 representative *BNAC* genes during cold, gibberellin and H_2O_2 stresses **(Figure C)**. The red standard curve represents reference genes and other blue curve represents *BNAC* genes.
