# Supplementary material for: Molecular Characterization and Expression Profiling of NAC Transcription Factors in Brachypodium distachyon L
Source: PLoS One. 2015 Oct 7;10(10):e0139794. doi: 10.1371/journal.pone.0139794 (PMC4596864; doi:10.1371/journal.pone.0139794)
Supplement: S2 File — Sequence LOGO view from 113 mature miRNA164 sequences. The height of the letter at each position represents the degree of conservation (Figure A). The predicted structure of the Bdi-miR164 synthetic precursors (Figure B). Mapping of BNAC mRNA cleavage sites. The red box represents target sequences of mature miR164. The cleavage sites are indicated by arrows (Figure C). (PDF) [file pone.0139794.s008.pdf]

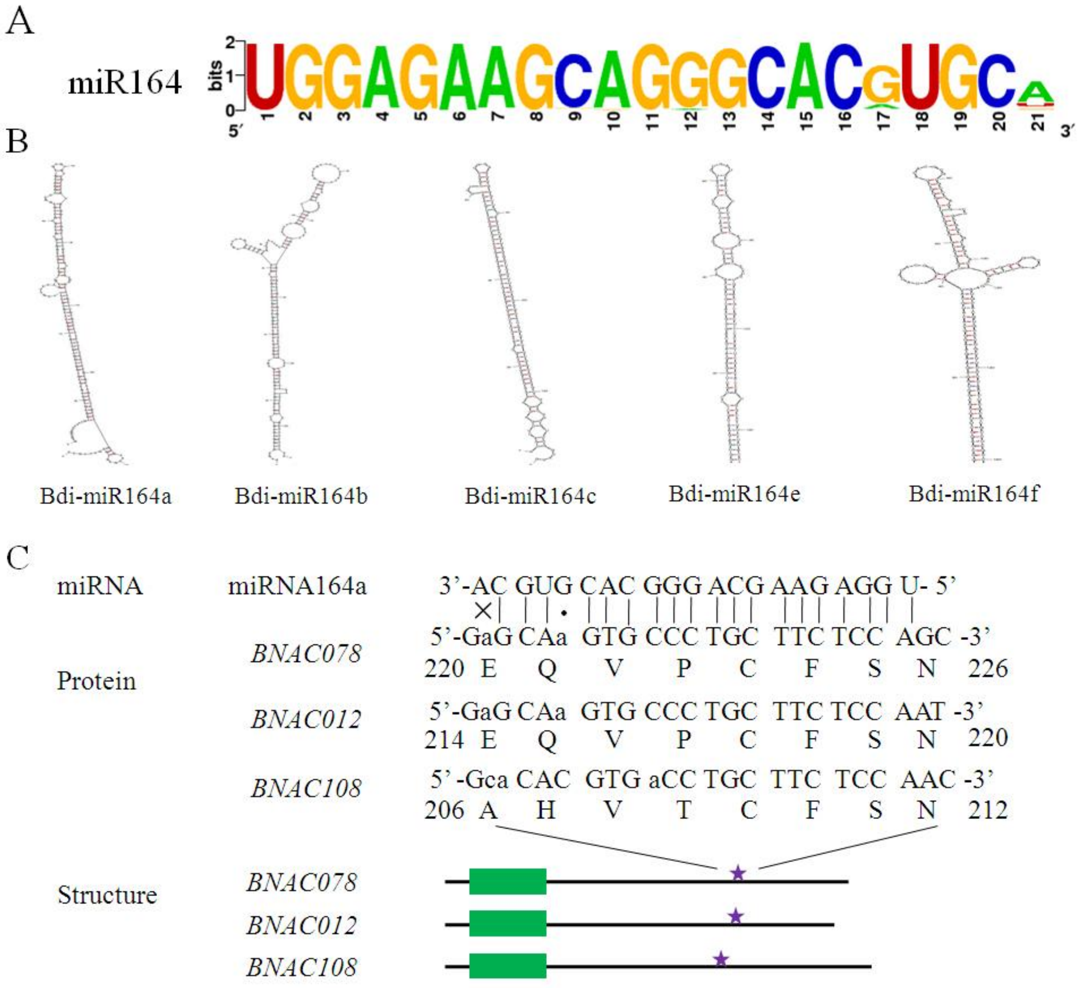

S2 File. Mature miR164 sequence analysis in plants and illustration of *BNAC* mRNA cleavage by Bdi-miR164. Sequence LOGO view from 113 mature miRNA164 sequences. The height of the letter at each position represents the degree of conservation **(Figure A)**. The predicted structure of the Bdi-miR164 synthetic precursors **(Figure B)**. Mapping of *BNAC* mRNA cleavage sites **(Figure C)**.
