# Supplementary material for: Molecular Characterization and Expression Profiling of NAC Transcription Factors in Brachypodium distachyon L
Source: PLoS One. 2015 Oct 7;10(10):e0139794. doi: 10.1371/journal.pone.0139794 (PMC4596864; doi:10.1371/journal.pone.0139794)
Supplement: S1 Table — (DOCX) [file pone.0139794.s009.docx]

**S1 Table. Primer sequences used for quantitative real-time RT-PCR.**

| **Gene** | **Primer name** | **Primer sequence** | **Temperature (℃)** | **product size (bp)** |
| --- | --- | --- | --- | --- |
| **BNAC002** | BNAC002-F | CAGGGGAAGAACATTCAGGA | 60 | 164 |
|  | BNAC002-R | AATTCGGCAAAGACACCAAC |  |  |
| **BNAC005** | BNAC005-F | GATGCGCTCGGATTATGATT | 60 | 210 |
|  | BNAC005-R | TTGGTCGGTTCATCATCAAA |  |  |
| **BNAC006** | BNAC006-F | TTGTGGAGCGAGACAATGAG | 60 | 214 |
|  | BNAC006-R | TCGGGTATTGGCTCAGACTT |  |  |
| **BNAC010** | BNAC010-F | GCCAAGCATTTCAGTGGTTT | 60 | 233 |
|  | BNAC010-R | GAAGCTGGCTCCTTCTCCTT |  |  |
| **BNAC017** | BNAC017-F | TAGCGGCAGTTCCTTCAAGT | 60 | 160 |
|  | BNAC017-R | TTGAAAGCAGAAGCCACCTT |  |  |
| **BNAC019** | BNAC019-F | CTGCTGCCATTTGCTTTGTA | 60 | 218 |
|  | BNAC019-R | CTTCACGTTCAGAGCAACCA |  |  |
| **BNAC022** | BNAC022-F | GGATCGAAATACGGAGTGGA | 60 | 184 |
|  | BNAC022-R | TGCCAGCATTCTCAACAAAG |  |  |
| **BNAC024** | BNAC024-F | TTTATTGGATTTGGGGTGGA | 60 | 191 |
|  | BNAC024-R | TCGAATGGGCCATAGAAAAC |  |  |
| **BNAC026** | BNAC026-F | CCCAGTTCTCATTTGGCAAT | 60 | 242 |
|  | BNAC026-R | GCTGTGCGCTGTGTATTCAT |  |  |
| **BNAC027** | BNAC027-F | TCATCAGCGACTTCCTTGACT | 60 | 205 |
|  | BNAC027-R | TGATGCTGATACTGCAGTTGG |  |  |
| **BNAC031** | BNAC031-F | CCCGATCTTCTCGCAGTCTC | 60 | 213 |
|  | BNAC031-R | GCTGGATTCAGGTGGAAGCT |  |  |
| **BNAC039** | BNAC039-F | CCAACAGATGTCGAGCTCGT | 60 | 211 |
|  | BNAC039-R | TGGCACGGTTTGTCCTAGAC |  |  |
| **BNAC049** | BNAC049-F | AAGGAGGACAACGACTGGTTC | 60 | 174 |
|  | BNAC049-R | CAAGATTGTTTGCCACATCCT |  |  |
| **BNAC067** | BNAC067-F | TCCTTCCAATGGACTCAGATG | 60 | 171 |
|  | BNAC067-R | GCTGGTCGTCGTCATAGAGAG |  |  |
| **BNAC070** | BNAC070-F | TCTGAAGAATGGGTGGTGTG | 60 | 212 |
|  | BNAC070-R | GCCTCTTCTTGCAGGTCATC |  |  |
| **BNAC073** | BNAC073-F | TGAGGAAGAAGGTAGCGTCAA | 60 | 167 |
|  | BNAC073-R | AGTGCCTGTCGGGTATTTCTT |  |  |
| **BNAC076** | BNAC076-F | CATGGGTCTCTCCTCCCTTAC | 60 | 172 |
|  | BNAC076-R | GAGGAAACCGTTGAAGGAAAG |  |  |
| **BNAC079** | BNAC079-F | TCAAAGTACGTGAAGGGTTGC | 60 | 192 |
|  | BNAC079-R | CATGACCCAGTCCGTCTTCT |  |  |
| **BNAC092** | BNAC092-F | TACAAGCTTCGCATTTTGCTT | 60 | 245 |
|  | BNAC092-R | CTTCTGCAGGGTCTGTGTTTC |  |  |
| **BNAC098** | BNAC098-F | CCTCAGCTTCAGTTCCAAGAA | 60 | 185 |
|  | BNAC098-R | GACTTTGACGATGCAGCAGAT |  |  |
| **BNAC105** | BNAC105-F | GATCTATACGCCGCCTAATCC | 60 | 238 |
|  | BNAC105-R | ATCATCTGAAACCTGGTGCTG |  |  |
| **BNAC106** | BNAC106-F | TTGGCCTGAAGAAGAAGTTCA | 60 | 185 |
|  | BNAC106-R | TATCACCCAGTTGCTGGATTC |  |  |
| **BNAC113** | BNAC113-F | TGGCAGCAATACAGTTCGGTC | 60 | 125 |
|  | BNAC113-R | AAGAGGAACCCGCAGTCGTC |  |  |
| **Ubi4** | Ubi4-F | TGACACCATCGACAACGTGA | 60 | 142 |
|  | Ubi4-R | GAGGGTGGACTCCTTCTGGA |  |  |
