# Supplementary material for: Molecular Characterization and Expression Profiling of NAC Transcription Factors in Brachypodium distachyon L
Source: PLoS One. 2015 Oct 7;10(10):e0139794. doi: 10.1371/journal.pone.0139794 (PMC4596864; doi:10.1371/journal.pone.0139794)
Supplement: S5 Table — (DOCX) [file pone.0139794.s013.docx]

**S5 Table.** **Critical amino acid sites of functional divergence between subgroups of the NAC family in *Brachypodium*.**

| **Subgroup** | **Subgroup** | **Type-I** | **Type-II** |
| --- | --- | --- | --- |
| CUC | TIP | 249N,444K | 242P |
| CUC | SNAC | 193V | 249N,380V |
| CUC | TERN | 190I,193V,194D,239F,240F,242P,243R,244D,247Y,248P,**249N**,377T,380V,**382H**,384Y,441P,443F,**444K** | 192E,**242P**,**247Y**,**248P**,**249N**,378D,**382H**,**444K** |
| CUC | X | 248P | 190I,193V,194D,242P,243R,244D,383E,444K |
| CUC | VIII | 190I,192E,193V,194D,239F,240F,242P,243R,244D,247Y,248P,249N,377T,378D,379W,380V,381M,382H,383E,384Y,441P,442V,443F,444K |  |
| CUC | IX | **247Y**,378D,382H | 193V,242P,243R,244D,**247Y**,248P,249N,377T,380V,382H,441P,443F |
| VND | TIP | 444K | 243R |
| VND | SNAC |  | 242P,243R,249N,378D,443F,444K |
| VND | TERN |  | 192E,242P,247Y,248P,249N,382H |
| VND | X |  | 190I,193V,194D,243R,244D,378D,383E,444K |
| VND | VIII | 190I,192E,193V,194D,239F,240F,242P,243R,244D,247Y,248P,249N,377T,378D,379W,380V,381M,382H,383E,384Y,441P,442V,443F,444K |  |
| VND | IX | 190I,192E,193V,194D,239F,240F,**242P**,**243R**,**244D**,**247Y**,248P,**249N**,377T,**378D**,379W,380V,381M,**382H**,383E,384Y,441P,442V,443F,**444K** | **242P**,**243R**,**244D**,**247Y**,**249N**,**378D**,**382**H,**444K** |
| VND | VII |  | 243R,382H |
| TIP | X |  | 383E |
| TIP | VIII | 378D |  |
| TIP | IX | 190I,192E,193V,194D,239F,240F,242P,243R,244D,247Y,248P,249N,377T,378D,379W,380V,381M,382H,383E,384Y,441P,442V,443F,444K |  |
| SNAC | X | 243R | 190I,193V,194D,242P,243R,244D,383E,444K |
| SNAC | VIII | 190I,192E,193V,194D,239F,240F,242P,**243R**,244D,**247Y**,248P,249N,377T,378D,379W,380V,381M,382H,383E,384Y,441P,442V,**443F**,**444K** | **243R**,**247Y**,**443F**,**444K** |
| SNAC | IX | 190I,192E,193V,194D,239F,240F,242P,243R,244D,247Y,248P,249N,377T,378D,379W,380V,381M,382H,383E,384Y,441P,442V,443F,444K |  |
| SNAC | VII |  | 243R,382H |
| TERN | X |  | 190I,192E,193V,194D,242P,243R,244D,247Y,248P,249N,378D,382H,383E,444K |
| TERN | VIII | 190I,192E,193V,194D,239F,240F,242P,243R,244D,247Y,248P,377T,378D,379W,380V,**382H**,384Y,441P,442V,443F,444K | **382H** |
| TERN | IX | 190I,192E,193V,194D,239F,240F,242P,243R,244D,247Y,248P,377T,378D,380V,382H,384Y,441P,442V,443F |  |
| X | VIII |  | 193V,194D,242P,247Y,383E,443F |
| X | IX |  | 194D,383E |
| X | VII |  | 190I,192E,193V,194D,242P,244D,247Y,249N,378D,382H,383E |

Sites in bold means they are responsible for both type-I and type-II functional divergence.
