# Supplementary material for: Molecular Characterization and Expression Profiling of NAC Transcription Factors in Brachypodium distachyon L
Source: PLoS One. 2015 Oct 7;10(10):e0139794. doi: 10.1371/journal.pone.0139794 (PMC4596864; doi:10.1371/journal.pone.0139794)
Supplement: S9 Table — (PDF) [file pone.0139794.s017.pdf]

S9 Table. Comparisons of phylogenetic classifications of *BNAC* genes

NACsubfamily (present work)	NAC subfamily in [12]	NAC subfamily in [25]	NAC subfamily in [63]
I (19)	NAC1 (6): 12, 62, 74, 78, 113, 117 NAM (13): 4, 5, 8, 9, 16, 27, 32, 69, 71, 94, 104, 111, 108	NAM|CUC3 (13): 4, 5, 8, 9, 16, 27, 32, 69, 71, 94, 104, 108, 111 NAC22 (2): 74, 113 NAC1 (4): 12, 62, 78, 117	NAC-d (19)
II (11)	OsNAC7 (10): 15, 19, 20, 30, 54, 58, 60, 73, 107, 114, 118	SND (10): 15, 19, 20, 30, 54, 58, 60, 73, 107, 114, 118	NAC-c (11)
III (12)	NAC2 (5): 39, 57, 75, 116, 93 ANCC011 (4): 31, 96, 97, 66 TIP (2): 21, 61 OsNAC8 (1): 36	TIP (12): 36, 39, 57, 75, 116, 93, 31, 96, 97, 66, 21, 61	NAC-b (12)
IV (14)	NAP (6): 6, 10, 26, 33, 35, 105, 106 OsNAC3 (3): 2, 22, 44, 49 ATAF (3): 40, 51, 85 SENU5 (0) AtNAC3 (0)	SNAC (14): 2, 6, 10, 22, 26, 33, 35, 40, 44, 49, 51, 85, 105, 106	NAC-a (14)
V (13)	ONAC22 (10): 3,11, 23, 29, 41, 52, 59, 68, 76, 92 TERN (3): 38, 50, 100	ANAC34 (10): 3,11, 23, 29, 41, 52, 59, 68, 76, 92 ONAC7 (3): 38, 50, 100	NAC-e (13)
VI (10)	—	ONAC1 (10): 18, 67, 70, 79, 80, 81, 82, 83, 84, 95	NAC-f (10)
VII (10)	—	ONAC3 (10): 42, 48, 64, 89, 90, 98, 99, 101, 102, 103	NAC-h (10)
VIII (9)	—	ONAC2 (9): 13, 24, 53, 55, 56, 65, 86, 87, 88	NAC-g (9)
IX (6)	—	ONAC3 (6): 1, 7, 37, 45, 47, 109	NAC-h (6)
X (8)	ONAC003 (8): 14, 17, 28, 34, 43, 46, 72, 110	ONAC4 (8): 14, 17, 28, 34, 43, 46, 72, 110	NAC-g (8)
XI (2)	—	ONAC5 (1): 25, 91	NAC-a (2)
XII (2)	—	ONAC3 (2): 77, 115	NAC-a (2)
XIII (1)	—	ONAC4 (1): 63	NAC-g (1)
XIV (1)	—	—	NAC-b (1)

[12] Ooka *et al*. **Comprehensive Analysis of NAC Family Genes in *Oryza sativa* and *Arabidopsis thaliana*.** DNA Res. 2003; 10: 239-247.

[25] Nuruzzaman *et al*. **Genome-wide analysis of NAC transcription factor family in rice.** Gene. 2010; 465: 30-41.

[63] Shen *et al.* **A Bioinformatic Analysis of NAC Genes for Plant Cell Wall Development in Relation to Lignocellulosic Bioenergy Production.** Bioenergy Res. 2009; 2: 217-232.
